# Scalable microfluidics for single-cell RNA printing and sequencing

**DOI:** 10.1186/s13059-015-0684-3

**Published:** 2015-06-06

**Authors:** Sayantan Bose, Zhenmao Wan, Ambrose Carr, Abbas H. Rizvi, Gregory Vieira, Dana Pe’er, Peter A. Sims

**Affiliations:** Department of Systems Biology, Columbia University Medical Center, New York, NY 10032 USA; Department of Biological Sciences, Columbia University, New York, NY 10027 USA; Department of Biochemistry & Molecular Biophysics, Columbia University Medical Center, New York, NY 10032 USA; Sulzberger Columbia Genome Center, Columbia University Medical Center, New York, NY 10032 USA

## Abstract

Many important biological questions demand single-cell transcriptomics on a large scale. Hence, new tools are urgently needed for efficient, inexpensive manipulation of RNA from individual cells. We report a simple platform for trapping single-cell lysates in sealed, picoliter microwells capable of printing RNA on glass or capturing RNA on beads. We then develop a scalable technology for genome-wide, single-cell RNA-Seq. Our device generates pooled libraries from hundreds of individual cells with consumable costs of $0.10–$0.20 per cell and includes five lanes for simultaneous experiments. We anticipate that this system will serve as a general platform for single-cell imaging and sequencing.

**Electronic supplementary material:**

The online version of this article (doi:10.1186/s13059-015-0684-3) contains supplementary material, which is available to authorized users.

## Background

A broad set of tools including microarrays [1], RNA-Seq [2], qRT-PCR [3], and RNA-FISH [4–6] now enables multiplexed, genome-wide, or targeted analysis of individual cells. Multiple schemes for transcriptome-wide library preparation have been tailored specifically to single-cell analysis [2, 7–11] and engineered for multiplexing [9, 12] and even mitigation of amplification bias [13]. Despite this progress, single-cell transcriptomics remains technically demanding and expensive, and there exists a need for simpler, more scalable approaches to RNA manipulation. Furthermore, the benefits of profiling hundreds or even thousands of individual cells in parallel from a single specimen for producing ‘cell censuses’ of organs and capturing the responses of rare subpopulations to stimuli are becoming increasingly clear [12, 14, 15].

Microfluidics is playing an increasingly important role in addressing the challenges of manipulating low-input RNA samples and allowing automated, parallel analysis of individual cells [3, 15–20]. Processing low-input and single-cell samples in microscale volumes reduces contamination and reagent consumption while increasing capture efficiencies [16, 18]. Multiple microfluidic platforms for single-cell qRT-PCR and RNA-Seq have been reported [3, 15, 18]. A commercial system from Fluidigm now allows routine, automated cDNA library preparation and pre-amplification from tens of individual cells in parallel [14, 15, 18].

Unlike systems used for population-level analysis of RNA from large bulk samples which employ solid-phase capture, most microfluidic systems capture RNA in solution, keeping the captured material confined by microscale chambers. Hence, when fluid exchange is required for multi-step enzymatic processing of RNA, the captured material must be transferred to a new microfluidic chamber using relatively complex devices [16, 17, 20]. In addition, reagents must be delivered to each chamber independently using individually addressable reagent flow systems for each sample. Solid-phase capture offers numerous advantages, including facile fluid exchange, removal of contaminants, and compatibility with high-resolution imaging. The ability to exchange reagents without physically moving the captured material also facilitates scalability and miniaturization because multiple chambers controlled by on-chip valves are not required to process an individual sample. Here, we report and characterize a scalable, high-density microfluidic system for solid-phase RNA capture on either glass coverslips or polymer beads. As an application of this platform, we demonstrate a low-cost, high-throughput technology for RNA-Seq of hundreds of individual cells in parallel.

## Results and discussion

### PDMS microwell flow cell for single-cell transcriptome capture

**Fig. 1:**
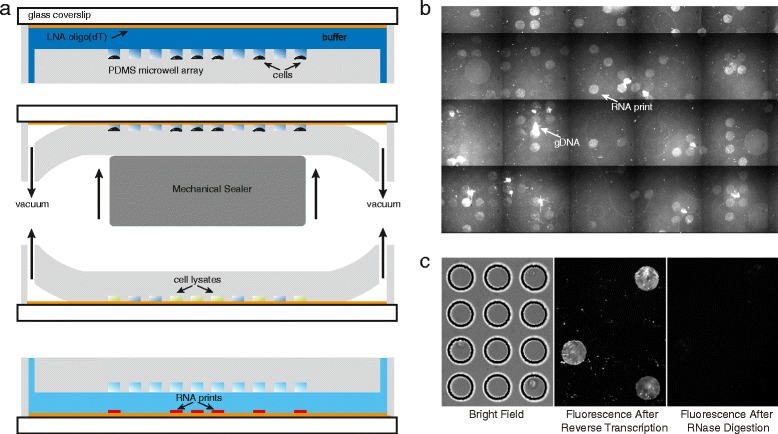
Schematic and fluorescence imaging data for single-cell RNA printing. **a** Cells are first deposited in the microwell array by gravity. The glass surface opposite the microwell array is covalently functionalized with oligo(dT) primers for mRNA capture (orange line). The device is then rapidly and conformally sealed against a glass surface in the presence of lysis buffer, flipped over, and held in a sealed position using negative pressure. Single-cell lysates (green) become trapped in the sealed microwells, and mRNA hybridizes to the oligo(dT) primers on the glass surface, resulting in single-cell mRNA ‘prints’ (red lines). **b** An array of single-cell mRNA prints on a glass coverslip generated using the device in Fig. 1a and imaged after on-chip reverse transcription. The double-stranded RNA/DNA hybrids are stained with SYTOX Orange, an intercalator dye and imaged on the glass surface. More than 96 % of the prints result from individual cells. Note that the bright spots in the image that are not registered with the array originate from genomic DNA aggregates that were not fully removed by DNase digestion. **c** Close-up images of single-cell RNA printing. The left panel is a bright field image of three cells in individual microwells of the array, the middle panel is a fluorescence image of the corresponding RNA prints on the glass surface after reverse transcription and staining with SYTOX Orange, and the right panel is a fluorescence image of the glass surface after RNase digestion, demonstrating that the fluorescent prints originate from captured RNA

**Fig. 2:**
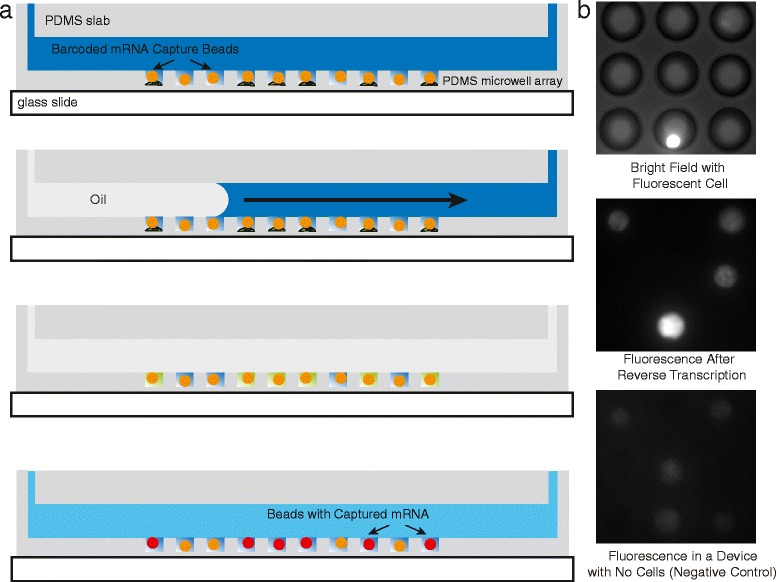
Schematic and fluorescence imaging data for single-cell RNA capture on beads. **a** For mRNA capture on polymer beads, the microwell array is fabricated in a thin PDMS layer on top of a glass slide or coverslip with a microfluidic flow channel above. Cells are first deposited in the microwell array by gravity followed by beads (while circles) covalently functionalized with oligo(dT) primers (orange circular outlines). A lysis buffer is introduced followed by rapid displacement of fluid in the channel with oil, which conformally seals the array. Single-cell lysates (green) become trapped in individual microwells and mRNA hybridizes to the oligo(dT) on the beads (red circular outlines). **b** Close-up images of single-cell RNA capture on beads. The top panel is a bright field/fluorescence overlay of a microwell array in which four microwells contain a bead, but only one contains both a bead and a cell (fluorescently labeled with live stain). The middle panel is a fluorescence image of the array after RNA capture, reverse transcription, and staining with SYTOX Orange. Note that the bead associated with a cell is significantly brighter than the other beads. The bottom panel is a fluorescence image of beads in an array from a negative control experiment involving no RNA or cells, showing that the beads have a certain level of background fluorescence in the presence of stain, which explains the majority of the background signal observed in the beads with no cell in the middle panel

Our microfluidic platform is comprised of a simple flow cell with an array of microwells embedded in either the top or bottom of the device similar to what we have reported previously for high-throughput DNA sequencing [21] and digital PCR [22]. We drive fluids through the flow cell manually at a standard laboratory bench by laminar flow using a syringe or pipette. Fluid exchange in the microwells occurs by diffusion, while cells and beads can be loaded by gravity. We fabricate the microwell arrays in polydimethylsiloxane (PDMS), a silicone rubber commonly used in soft lithography [23]. PDMS allows inexpensive, rapid, and repeatable fabrication from molds produced on silicon in photoresist using standard photolithography [23]. In addition, the material properties of PDMS, including its hydrophobicity and flexibility, facilitate reversible sealing of the microwells against a flat surface using mechanical deformation and negative pressure [21, 24] (Fig. 1a) or introduction of oil [25] by laminar flow (Fig. 2a). Several variations on microwell arrays have been reported previously for gene-specific analysis in individual cells [26], targeted analysis of gene panels [27], or paired chain analysis of the antibody repertoire [28]. Here, we have advanced this technology for genome-wide RNA capture and sequencing.

Our device is capable of solid-phase capture of RNA from individual cells via two modes of operation: RNA ‘printing’ on glass and RNA capture on beads. The overall approach is reminiscent of previously reported ‘microengraving’ systems for capturing specific protein secretions from individual cells [29]. In RNA printing mode (Fig. 1a), individual cells are loaded in the microwells, which are fabricated in a PDMS slab that faces a glass coverslip. Oligo(dT) primers are covalently grafted to the glass surface so that mature mRNA molecules can be immobilized by hybridization of their poly(A) tails. Immediately following the introduction of lysis buffer, we seal the microwells by mechanically placing them in conformal contact with the functionalized glass surface. Cell lysis releases mRNA into the solution confined by the microwells, resulting in hybridization to the oligo(dT)-coated glass coverslip. By placing the flow channel under negative pressure, the seal can be maintained in the absence of mechanical force, making the device transportable and readily accessible to an optical microscope [21].

Because the mRNA is immobilized on a glass surface, enzymatic processing steps can take place on-chip, simply by sequential flow of reagents through the device. After incubating the trapped, single-cell lysates with the glass capture surface, we release the seal and vigorously rinse the flow cell with a detergent-containing buffer followed by a reaction mixture containing DNase. Because the oligo(dT) primers comprise locked nucleic acid (LNA) [30], they are resistant to nuclease digestion. The immobilized single-cell mRNA libraries are then reverse transcribed in parallel, and the resulting mRNA/cDNA hybrids can be visualized by fluorescence microscopy after staining with a fluorogenic intercalator dye. Figure 1b shows a fluorescence image of single-cell transcriptome ‘prints’ arrayed on a glass coverslip as described above.

We conducted a simple control experiment to verify that the printed material does, in fact, originate from RNA. While there are some imperfections, including aggregates of genomic DNA that were not fully digested (but are reduced in intensity by DNase treatment), we can show that the vast majority of material imaged in the circular prints originates from RNA. The left panel of Fig. 1c shows a bright field image of a microwell array in which three microwells each contain an individual cell. The resulting RNA prints (middle panel) that can be visualized after reverse transcription are ablated by incubating the surface with RNaseH (right panel), which selectively digests RNA in RNA/DNA hybrids. Conversion of RNA/DNA hybrids to single-stranded cDNA precludes detection using the intercalator dye, and so removal of RNA from the prints eliminates the fluorescence signal almost completely. We note that Fig. 1c also contains some small fluorescent objects associated with the interstitial walls of the microwell array or with microwells that did not contain a cell. These are substantially reduced in intensity by RNase treatment, confirming that they are, in fact, RNA that is spuriously captured or non-specifically adsorbed. These objects could arise due to contamination from dead cells or other sources of freely floating RNA introduced with the cells prior to sealing. Nonetheless, the vast majority of the observed signal in Fig. 1c is associated with the circular mRNA prints that correlate perfectly with microwells that initially contained a cell.

Figure 2a shows a second, very similar version of the device where the microwells are fabricated in PDMS on a glass slide, and the sealing is accomplished by laminar flow of oil. Using nearly the same procedures as described above for RNA printing mode, we use this version of the device to capture RNA on beads. After introducing cells, we can load beads into the microwells by gravity and achieve super-Poisson loading by using beads with a mean diameter greater than the radius of the microwells. Like the glass surface in Fig. 1a, we coat the beads in oligo(dT) to facilitate mRNA capture after cell lysis and sealing. Figure 2b shows bright field and fluorescence images of a bead-containing microwell array loaded with individual cells following solid-phase mRNA capture and reverse transcription. The bead contained in a microwell that also contains a cell is substantially more fluorescent following reverse transcription than the other beads. While there is some fluorescence signal associated with beads that do not contain a cell, this is mainly due to non-specific staining of the high-density of single-stranded primers on the bead surface and non-specific staining of the bead itself, as shown in the third panel of Fig. 2b where we depict fluorescence images of beads in the absence of cells, cell lysate, or RNA as a negative control.

### A scalable platform for single-cell RNA-Seq

To demonstrate the potential of our system for single-cell transcriptomics, we have developed a scalable platform for single-cell RNA-Seq based on the bead capture modality of our device. The low reagent volumes required for microfluidic processing result in a significant cost reduction relative to conventional methods [18]. However, a further reduction in cost can be realized by using microfluidics in combination with recently reported schemes for cDNA barcoding, such as the CEL-Seq strategy [9]. By introducing a cell-specific barcode to the cDNA during reverse transcription, all subsequent sequencing library preparation steps can be accomplished on pooled cDNA from multiple cells, further reducing hands-on time and reagent consumption. This approach has already been realized on a large scale in combination with automated liquid handling robots [12]. Here, we describe a microfluidic implementation of this approach.

**Fig. 3:**
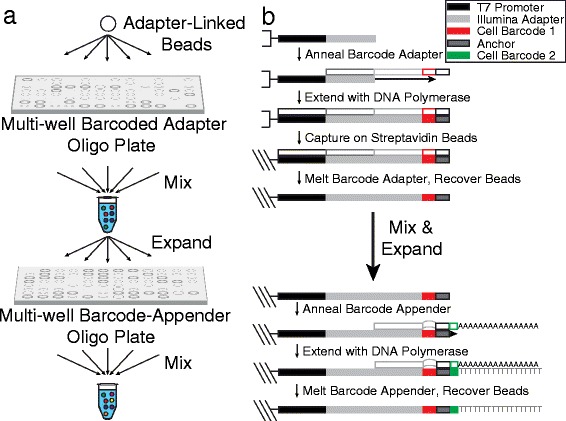
Combinatorial scheme for synthesis of barcoded capture beads. **a** Beads are first attached to a set of barcoded oligonucleotides in a multi-well plate, pooled into a single tube, and then re-distributed into a second multi-well plate for combinatorial addition of a second barcode sequence and capture site (oligo(dT)). **b** Detailed molecular biology for solid-phase, combinatorial barcode synthesis. A first barcode sequence is copied onto a dual-biotinylated oligonucleotide containing the T7 promoter sequence and a partial Illumina adapter using DNA polymerase. The resulting double-stranded DNA is conjugated to streptavidin-coated beads, and the non-biotinylated strand is removed. After pooling and expanding the beads, a second reaction is used to add a second barcode sequence and oligo(dT) by priming off of a universal anchor sequence that follows the first barcode

We generated a pool of mRNA capture beads in which each bead is attached to approximately 1 billion copies of a primer terminated on the 3′-end with one of 960 possible barcode sequences followed by oligo(dT) using a combinatorial synthesis technique (Fig. 3). If 100 of the cells loaded into the microwells of our device receive a random barcoded bead from the pool, we expect the mRNA from approximately 90 of them to be uniquely labeled based on the binomial distribution. A copy of the T7 promoter sequence (TPS) and part of an Illumina sequencing adapter (ISA) comprise the 5′-end of the capture primer (Additional file 1: Table S1) to allow linear pre-amplification by *in vitro* transcription (IVT) and library enrichment by PCR (Fig. 3b). To create this large pool of barcoded beads, we first copy 96 different barcode-containing oligonucleotides (Additional file 1: Table S2) onto a dual-biotinylated oligonucleotide containing TPS and ISA by primer extension with DNA polymerase in a 96-well plate. Each barcode is terminated with a universal, 6-base anchor sequence that becomes the 3′-end of the biotinylated oligonucleotide after the first round of primer extension (Fig. 3b). After this first reaction, we immobilize each barcoded oligonucleotide on a set of streptavidin-coated Sepharose beads, quench the reaction, combine all of the barcoded beads in a pool, and remove original barcode-containing strand by denaturation. At this point, the pool of beads is split into 10 new reactions and each containing one of 10 unique second barcodes along with poly(dT) (Additional file 1: Table S3) are added to the 3′-end of the immobilized oligonucleotide by primer extension from the universal anchor sequence (Fig. 3b). After quenching this reaction, we again pool the beads, remove the unbiotinylated strand, and wash. The resulting pool of beads contains 960 barcoded capture primers.

**Fig. 4:**
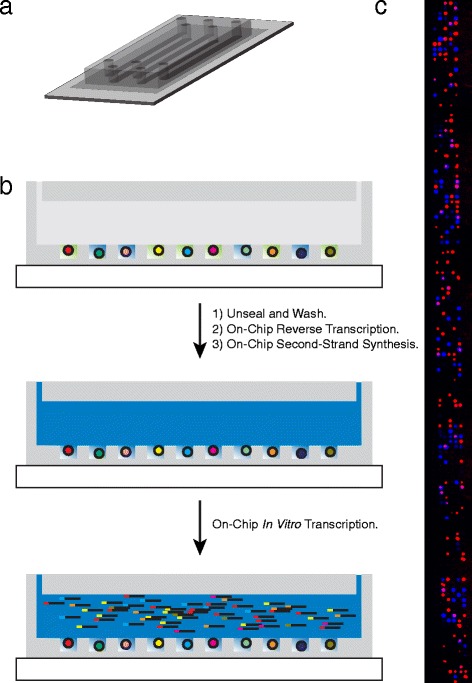
Flow cell device for single-cell RNA-Seq. **a** Graphical representation of our five-lane microwell array flow cell device for single-cell RNA-Seq. **b** Schematic of on-chip steps for single-cell RNA-Seq. After depositing cells, barcoded capture beads (barcode sequences represented as different colors), and sealing as in Fig. 2a, single-cell lysates (green) are trapped in individual microwells and mRNA hybridizes to the barcoded capture beads. The device is unsealed and rapidly washed by flow before on-chip, solid-phase reverse transcription and second-strand synthesis followed by elution and pre-amplification of the pooled library by *in vitro* transcription. **c** Montage of fluorescence images from part of one lane of the device in (**a**) showing beads (red) and cells (blue) loaded in the array. Note that this image was acquired following cell lysis while the device is sealed, and so the blue live stain fills the entire volume of the corresponding microwell and is confined to the microwell by sealing

We constructed a PDMS microwell device containing five flow channel lanes for physical multiplexing of samples and >10,000 microwells (Fig. 4a). The cylindrical microwells are 50 μm in diameter and height with a volume of <100 pL. Cells are loaded in individual microwells randomly, according to Poisson statistics, such that the majority of cell-containing wells contain one cell. We tune the concentration of our cellular suspension to avoid overloading the microwell array. Specifically, if we capture approximately 100 cells in every 1,000 microwells of a given array, then <5 % of microwells will contain more than one cell. We then load beads into the wells at a somewhat higher density because the mean diameter of the beads (approximately 30 μm) significantly reduces the probability double-loading (Fig. 4bc). While we occasionally observe microwells with more than one bead or more than one cell, size constraints make it rare to observe both beads and cells in an overloaded microwell. Given our pool of 960 cell-identifying barcodes and five lanes, the capacity of this system for single-cell RNA-Seq is approximately 600 cells at a unique barcoding rate of >94 %. We can scale our system and increase capacity simply by synthesizing additional barcodes and/or adding microwells to our device.

After loading the cells and barcoded beads, we use the procedures described above to trap single-cell lysates in sealed microwells, immobilize captured mRNA on beads, and reverse transcribe (Fig. 4b). Following on-chip second-strand synthesis, we simultaneously elute and pre-amplify our pool of single-cell libraries overnight by IVT using T7 RNA polymerase (Fig. 4b). We then remove the resulting amplified RNA (aRNA) from each lane using a pipette, reverse transcribe the aRNA from each lane with primers containing lane-identifying barcodes, pool the cDNA libraries from all five lanes, and enrich the sequencing library in a single PCR reaction. The primers used for aRNA reverse transcription contain 8-base unique molecular identifiers (UMIs) so that the vast majority of cDNA molecules are distinguishable. That way, genes can be quantified from sequencing data based on the number of UMIs associated with each gene rather than the number of reads, mitigating noise and bias that result from exponential amplification by PCR [31, 32].

### Demonstration and analysis of highly multiplexed single-cell RNA-Seq

We used our microfluidic device to obtain RNA-Seq profiles from approximately 600 cells across five lanes from two commonly used human cancer cell lines. We refer to this run of our device as Experiment 1 throughout the text. One of the five lanes contained U87 human glioma cells, one contained MCF10a human breast cancer cells, and the other three contained a mixture of both cell lines. These two cell lines are highly mesenchymal, have been cultured for numerous passages, and have relatively similar expression profiles. Nonetheless, they are distinguishable by a few key genes and can be readily separated in our dataset. In addition, we used a slightly different protocol with less expensive reagents to obtain profiles of approximately 500 cells across five lanes for a different cell pair (U87 cells and the diploid cell line WI-38, which has not undergone malignant transformation) in Experiment 2. Importantly, in both experiments, we do not obtain high-quality data from all of the cells introduced into our device. In both experiments, 30–50 % of single-cell profiles exhibit very low coverage, which could be due to a number of factors including cell viability, the rare occurrence in which a cell is paired with two beads, and incomplete cell lysis. Hence, despite achieving higher throughput than previously reported microfluidic systems, we have considerable room for improvement in terms of fractional yield.

One major concern with any pooled library scheme is cross-talk between cell-identifying barcodes. Here, we addressed this issue by quantitative analysis of Experiment 1. Although our device is sealed during cell lysis and RNA capture, any imperfections in sealing and washing could lead to inter-well contamination as demonstrated in Figure S3 in Additional file 1. We addressed and quantified our cross-talk using both sequencing and imaging data. Because our device is compatible with fluorescence microscopy, we labeled a fraction of the streptavidin molecules on each bead with red-fluorescent AlexaFluor 647 and pre-stained the cells with a blue-excitable live stain. This allowed us to quantify the number of cells successfully paired with a barcoded capture bead and estimate the number of barcodes we expect to observe in our sequencing data for each lane. The sequencing data revealed that more barcodes were present in our library for each lane than expected based on our imaging data. Careful inspection revealed that the number of molecules associated with a given barcode placed the barcodes in two distinct populations (Figure S1ab in Additional file 1). The size of the population of barcodes associated with a larger number of molecules was highly consistent with our imaging data (within approximately 8 %), which we take to demarcate our single-cell RNA-Seq profiles (Figures S1c, S2 in Additional file 1). The second, larger population of barcodes with relatively few associated molecules likely results from multiple potential sources including sequencing error, actual cross-talk or spurious capture within our microfluidic device, and PCR jumping as observed in other implementations of multiplex single-cell RNA-Seq [12]. Across all five lanes the cell-identifying barcodes that we did not associate with actual cells in our device had 200–300× fewer molecules per barcode than those associated with cells (based on the ratio of median unique molecules in the two populations).

**Fig. 5:**
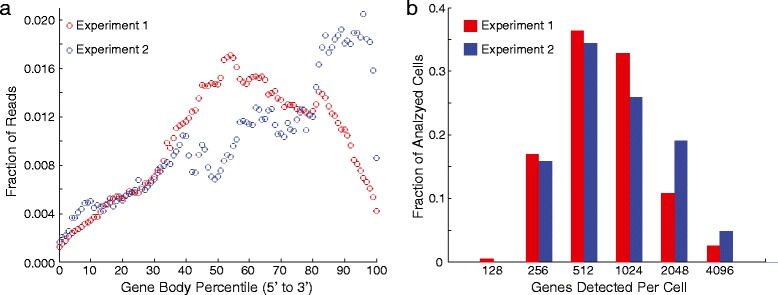
Analysis of single-cell RNA-Seq data. **a** Gene body distribution for uniquely mapped reads showing that we are primarily sequencing the 3′-end of transcripts, as expected. **b** Histogram of the number of genes detected per cell for the 396 single-cell profiles used in all subsequent analysis of Experiment 1 and 247 single-cell profiles used in all subsequent analysis of Experiment 2

To demonstrate that our device is actually producing useful single-cell RNA-Seq profiles, we examined several key metrics. Our library preparation protocol is based on CEL-Seq [9] where, rather than sequencing the full gene body and normalizing by transcript length, the 3′-end of transcripts are sequenced and counted. Figure 5a shows the expected distribution of mapping positions for 3′-end sequencing, with most reads mapping to the 3′-UTRs or coding sequences. Subsequent analysis to demonstrate cell type separation using our dataset will rely on the 396 single-cell profiles that we obtained with the highest coverage. Although we detect only 635 genes on average across all cells profiled in Experiment 1, we detect an average of 876 genes from the top 396 cells (Fig. 5b). Hence, the 204 cells that we discard from subsequent analysis have an average of approximately 170 genes detected per cell. Similarly, for Experiment 2, we detect an average of 1,030 genes from the top 247 single-cell profiles (Fig. 5b), but only approximately 530 genes on average across all cells. Despite these shortcomings, recent studies have demonstrated cell or phenotype separation from low coverage single-cell RNA-Seq data [12, 15]. Indeed, our detection efficiency is at least comparable if not better than previously reported methods for large-scale single-cell RNA-Seq using pooled barcode library preparation, where detection of hundreds of molecules per cell was reported [12] (whereas we are reporting detection of hundreds of genes).

**Fig. 6:**
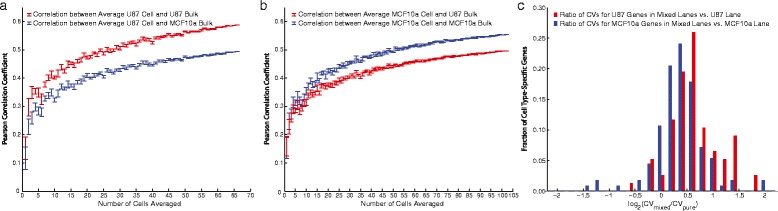
**a** Comparison of single-cell median and population-level RNA-Seq profiles for cells originating from the U87-exclusive lane in Experiment 1. Each data point was obtained by constructing a median profile from a given number of cells and repeating this 10 times by random sampling with replacement to obtain a median Pearson correlation coefficient and error bar (SEM). This exercise was repeated for comparison to both the U87 and MCF10a bulk RNA-Seq profiles to demonstrate better concordance between the U87 single-cell profiles and the U87 bulk profile. **b** Same as (**a**), but for single-cell profiles in the MCF10a-exlusive lane. **c** We conducted differential expression analysis to obtain cell type-specific gene sets for the U87 and MCF10a cells based on single-cell profiles from the pure-cell lanes. Here, we show a histogram of log-ratio of the coefficients of variation (CVs) for the cell type-specific gene sets between the mixed lane profiles and the profiles from the respective pure lanes. As expected, the heterogeneity given by CV is greater for cells in the mixed lanes than in the cell type-exclusive lanes for the cell type-specific genes

To assess the similarity of our single-cell expression profiles to conventional, population-level RNA-Seq, we calculated the Pearson correlation between bulk RNA-Seq and single-cell medians constructed from different numbers of individual cells after normalizing by the total number of molecules detected in each cell (Fig. 6a, b). We conducted this analysis on single-cell profiles originating from the U87-exclusive and MCF10a-exclusive lanes in Experiment 1, randomly sampling the complete sets of profiles 10 times without replacement for each point in the curves shown in Fig. 6a and b. This analysis shows that the single-cell medians constructed from U87 single-cell profiles correlate better with the bulk U87 RNA-Seq profile than with the bulk MCF10a RNA-Seq profile (Fig. 6a) and vice versa (Fig. 6b). It also shows that the single-cell median correlations saturate around r = 0.55–0.60 depending on the cell type. As a point of comparison, a similar analysis has been reported for CEL-Seq and DR-Seq, which are closely related protocols with implementations that have not been scaled using microfluidics. These two approaches gave population-level Pearson correlations of 0.71 and 0.69, respectively [33]. We note that, although our correspondence with population-level RNA-Seq is somewhat worse than what has been reported previously, the datasets used for comparison in our case are not direct technical replicates taken from the same sample of cells that was used for single-cell RNA-Seq (and the MCF10a profile was obtained from a public source and acquired by a different laboratory) [34].

To further demonstrate the robustness of our dataset, we attempted to build a classifier for U87 and MCF10a cells in Experiment 1. We used the single-cell profiles from the lanes that contained either exclusively U87 cells of MCF10a cells to identify 189 differentially expressed genes (*P* <0.05, Wilcoxin rank-sum test). We expect that the three lanes containing a mixture of individual cells will be globally more heterogeneous with respect to expression of the U87-specific and MCF10a-specific genes identified by this analysis. Figure 6c shows the log-ratio of the coefficients of variation (CVs) for each of these two genes sets between the mixed lane profiles and the profiles from the respective pure lanes. As expected, the log-ratio of CVs is greater than zero (CV ratio greater than one) for 92 % of U87-specific genes and 85 % of MCF10a-specific genes.

**Fig. 7:**
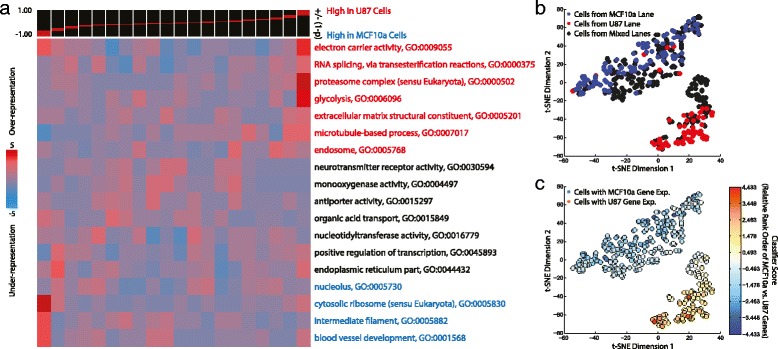
Cell type separation by single-cell RNA-Seq. **a** iPAGE gene ontology/pathway analysis based on rank-ordering of differentially expressed genes using +/−(1-p) where p is the *P* value for differential expression between the U87- and MCF10a-exclusive lanes given by the Wilcoxin rank-sum test. Values are positive for genes more highly expressed in U87 and negative for genes more highly expressed in MCF10a. **b** t-SNE clustering of 396 single-cell profiles based on the differentially expressed genes color-coated by the lane-of-origin of each profile. Two clear spatial clusters form and each is predominantly associated with a specific cell type-exclusive lane. **c** The same t-SNE clustering shown in (**b**) but color-coated with a score indicating expression of the U87-specific genes vs. the MCF10a-specific genes. The score is based on the relative rank ordering of U87- and MCF10a-specific genes in each cell (see Methods)

Figure 7a shows a pathway analysis of gene ontologies enriched across >11,600 genes that were both detected across our 396 single-cell profiles and available in the iPAGE database and ranked based on differential expression [35] in Experiment 1. We generated a matrix of Spearman correlation coefficients across our 396 profiles based on rank-ordering the 189 differentially expressed genes in each cell. We then clustered the data spatially using the t-stochastic neighborhood embedding (t-SNE) algorithm [36], a powerful clustering algorithm that has recently been applied to high-dimensional single-cell analysis data (Fig. 7b, c) [37]. Our t-SNE result contains two closely associated clusters of individual cells. To understand the origin of these two clusters, we displayed our t-SNE clustering with two different color-schemes. In Fig. 7b, we show how single-cell profiles from the various lanes of our device are distributed. As expected, one of the two clusters contains all of the cells from the MCF10a-exclusive lane, while the other contains nearly all of the cells from the U87 lane with a few exceptions. Single-cell profiles from the mixed lanes are distributed throughout the two clusters, although not with perfect uniformity. While the single-cell profiles from mixed lanes are distributed uniformly throughout the MFC10a cluster, there is some separation between a subset of mixed lane profiles and the U87-exclusive lane profiles in the U87 cluster. In Fig. 7c, we display the same clustering result with a different color scheme that indicates the relative rank ordering of the U87 vs. MCF10a gene sets in each profile. This metric clearly associates the cells in each of the two clusters with the expected cell type-specific expression pattern. However, the subset of the cells from the mixed lane in the U87 cluster that are somewhat separated from those in the U87-exclusive lane exhibit more ambiguous gene expression than others. This phenomenon could arise from one or some combination of several sources including: (1) a lane-specific batch effect; (2) the occasional presence of more than one cell in a microwell containing a single bead; (3) the association of a single cell-identifying barcode with more than one cell (which will occur for a few percent of cells given the current size of our barcode library); (4) low levels of cross-talk between wells or imperfections in the sealing of our device at specific locations in the microwell array; and (5) actual phenotypic differences between U87 cells in the presence of other U87 cells vs. MCF10a cells, which may secrete distinct and stimulatory diffusible factors. We also note that a small subpopulation of U87 cells in the U87-exclusive lane actually cluster with the MCF10a cells, highlighting the possibility of phenotypic heterogeneity within the U87 population. We conducted a similar differential expression and clustering analysis for Experiment 2 (Additional file 1: Figure S4) with closely related results. In any case, while there is certainly substantial opportunity to improve this technology from several angles, this analysis provides a compelling demonstration of the initial capabilities of this technology.

## Conclusion

Both versions of the microfluidic platform described above are, in principle, compatible with transcriptome-wide analysis of individual cells by RNA-Seq. Either could be combined with a sequence-based barcoding scheme to generate a pooled cDNA library from hundreds or thousands of individual cells. In the bead capture device, barcoding is not strictly necessary because physical means could be used to extract the beads from the microwells for downstream processing with conventional labware. Alternatively, fluorescently labeled oligonucleotide probes could be used to image captured RNA molecules similar to RNA-FISH. Probes could be introduced sequentially, imaged, and removed in cycles or combined with previously reported multiplexing schemes [6, 38, 39]. Similarly, sequential rounds of qRT-PCR in sealed microwells could allow targeted detection of specific genes or mutations in captured RNA.

Our system shares several features with a recently reported technology for massively parallel expression profiling, including the use of a microwell array and capture beads labeled with cell-identifying barcodes [27]. However, there are several crucial distinctions and advantages of our approach. Importantly, our microwell array device is constructed in such a way that it can be reversibly sealed during cell lysis and RNA capture. Significant loss of RNA occurs in our arrays when cells are lysed in unsealed or even imperfectly sealed arrays due to rapid diffusion of RNA molecules (Figure S3 in Additional file 1). In the context of the bead capture and RNA-Seq experiments, this could result not only in reduced RNA capture, but also significant cross-talk. We take advantage of the physical properties of PDMS, namely its flexibility and hydrophobicity, for high-fidelity, reversible sealing which is difficult to achieve using the agarose hydrogel device reported previously [27]. In addition, while the previous study reports targeted amplification of tens of genes in specific gene panels [27], we demonstrate genome-wide single-cell RNA-Seq with our system. Finally, our single-cell capture and pooled library preparation scheme costs $0.10–$0.20/cell even at a relatively modest scale of several hundred cells per run (see Additional file 1: Table S7), compared to the < $1/cell estimated at the 10,000-cell scale for this alternative approach [27]. This may, in part, be due to our approach of conducting several key steps in our pooled library preparation on-chip in a microfluidic channel, which allows us to use small reagent volumes. Hence, we anticipate a significant scalability advantage in terms of reagent costs.

The platform described by Fan *et al.* also has several advantages over our system. For example, Fan *et al.* use a much larger cell-identifying barcode set and a correspondingly larger microwell array, giving them higher throughput in terms of numbers of cells. In addition, they use beads that are highly monodisperse and better matched to their microwell size than in our current system. This allows them to effectively saturate their microwell array with beads and profile a larger fraction of captured cells. By implementing some of these features in the system described here, we could achieve higher throughput in terms of cell numbers.

Another shortcoming of our technology in its current implementation is the potential for batch effects across lanes of our device. The use of multiple lanes is useful in certain situations for conducting parallel experiments under multiple conditions. For example, one might introduce samples of identical populations into each lane, but subject the cells in each lane to a different chemical stimulus. However, in experiments where this is not necessary, it may be advantageous to increase the size of our barcoded bead pool and process all of the cells in a single large lane to minimize batch effects. Additionally, we could introduce spike-in standards (for example, ERCC spike-ins) to the beads, which would be processed alongside of any captured mRNA. Because these standards are not subject to variable capture efficiency due to lane- or cell type-specific differences in lysis efficiency and cover a broad range of lengths and sequence-space, we may be able better understand and even correct for the observed batch effects.

The advent of next-generation sequencing, imaging, and flow-based technologies have resulted in an explosion in high-dimensional single-cell analysis. While these unprecedented readouts have dramatically increased the numbers and types of observables available with single-cell resolution, similar advances in sample preparation and manipulation are required to fully realize the potential of these new tools. As shown here, the combination of pooled library preparation and microfluidics can make sequencing costs, which are plummeting rapidly, the limiting factor in determining scalability and throughput. Taken together with the potential for on-chip imaging and experimentation, microfluidic systems hold great promise for biological and biomedical applications of large-scale single-cell analysis.

## Methods

### Fabrication PDMS microwell arrays for single-cell RNA printing

Silicon wafer masters (approximately 4 in) with cylindrical pillars (diameter 50 micron; height 30 micron) for photolithography were obtained from Stanford Microfluidics Foundry and were subsequently exposed to 1H,1H,2H,2H-perfluorooctyltrichlorosilane (Alfa Aesar) vapor under vacuum for approximately 30 min to avoid curing of the PDMS on the wafer. PDMS (Sylgard 184, Dow Corning) was thoroughly mixed 9:1 (base:curing agent) and degassed under house vacuum for 2 h. Approximately 15 g of degassed PDMS was poured onto the 4 in silicon wafer master and allowed to cure overnight at approximately 90 °C. This slab with microwells was then gently peeled off from the master and used to construct PDMS microreactor flow cells.

### Surface chemistry on glass coverslip

VistaVision Microscope cover glass (22 × 50 × 0.16 mm) was plasma sterilized (Harrick Plasma) for approximately 5 min, and immediately immersed in 10 % acetic acid (pH 3.5) ethanol solution containing 0.5 % trimethoxysilanealdehyde (United Chemical Technologies), and incubated for 15 min. The cover glass was then washed with ethanol, air-dried and heat cured at 90 °C for 10 min. A 2.5 μM solution of 5′-aminated-LNA-oligo(dT) (Exiqon) in cyanoborohyride coupling buffer (Sigma) supplemented with 1 M NaCl was added on the aldehyde surface of the cover glass. The cover glass was incubated for 3 h at room temperature inside a humid chamber, and then washed with DI water. The aldehyde surface was then incubated in 10 % ethanolamine in cyanoborohydride coupling buffer for 30 min to quench the unreacted aldehydes.

### Construction of the flowcell

A rectangular slab (3.5 × 1.5 × 0.1 cm) of PDMS containing the microwell array in the center was cut and a double-sided adhesive tape (approximately 120 micron thickness, Grace BioLabs) was adhered to the flat side of the PDMS slab that contained the microwells. The tape was cut in an elongated hexagonal shape, which formed the microchannel in the flowcell. The other side of the tape was pasted on the LNA coated cover glass to build the microfluidic device. Two holes were punched at the two end of the microchannel with a biopsy punch, which acted as the inlet and outlet of the device and tubing were attached to allow liquid flow. The periphery of the PDMS slab was sealed on to the cover glass using epoxy glue.

### Experimental procedure for single-cell mRNA printing on glass

A suspension of U87 cells in PBS was flowed in to the device and loaded into the microwells by gravity (kept upside down) for 5 min at room temperature. After washing with PBS buffer supplemented with SUPERaseIN (Ambion), the microwells were sealed using an automated mechanical device by placing the flow cell upside down on a screw mounted on a motorized z-stage (ASI) so that the top PDMS slab containing the microwells was pressed against the glass bottom. After sealing the wells mechanically, the seal was retained by hermetic sealing to trap the single-cell lysate within a single microwell. The cells were lysed by freeze-thaw. Once the cells lysed, the mRNAs were captured on the LNA surface by hybridization of the 3′-polyA tail of the mRNA to the LNA-oligo(dT) during a 60 min incubation. The microwells were then unsealed and the flow cell was immediately and vigorously washed with the Wash Buffer (20 mM Tris pH 8.0, 50 mM NaCl, 0.1 % Tween-20), supplemented with SUPERaseIN (Fig. 1a). The flowcell was then incubated with TURBO DNase (Ambion) in TURBO DNase buffer, supplemented with 0.1 % Tween-20 and SUPERaseIN for 30 min at 37 °C to digest any residual genomic DNA. The mRNA captured on the LNA surface was reverse transcribed using M-MuLV Reverse Transcriptase (New England Biolabs) for 2 h at 42 °C in 1× M-MuLV Reverse Transcriptase buffer, supplemented with 10 mM DTT, 5 mM dNTPs, 0.1 % Tween-20 and SUPERaseIN. After reverse transcription the double stranded RNA-cDNA hybrids were stained with 10 nM SYTOX Orange dye (Invitrogen), an intercalator that is selective for double-stranded DNA, and incubated for 5 min prior to imaging.

The epifluorescence imaging system was constructed on an inverted Nikon Eclipse Ti-U microscope with 20×, 0.75 NA air objective (Plan Apo λ, Nikon). SYTOX Orange was excited using a 532 nm diode-pumped solid state laser (Dragon Lasers), and the fluorescence was collected and imaged onto an electron multiplying charge coupled device (EMCCD) camera (iXON3, Andor Technologies). The images were acquired with 0.5 s exposure time (controlled by external shutter) at 1 MHz digitization (with no EM gain). Automated scanning of the surface (motorized X-Y stage, ASI), image acquisition, and illumination were controlled with custom software written in C/C++. The images were analyzed using ImageJ software.

### Microfluidic device for single-cell RNA-Seq

For the single-cell RNA-Seq experiment we designed a monolithic PDMS based multi-channel device, by fabricating each channel with a microwell array. We used two key soft lithography techniques to fabricate this device. First, instead of using silicon wafer master directly for fabricating the microwell array as done in the case of RNA printing device, we generated a secondary master made out of PDMS. We did this because the aspect ratio of the micropillars results in a relatively fragile silicon master. We found the PDMS master to be more durable. Second, instead of using a double-sided adhesive tape for the device assembly, the bottom and the top of the device were bonded together by partial curing. This provided us with more durable and reliable partitions between the individual channels of our device than could be generated using tape. For the multi-lane microfluidic device, two different silicon wafer masters were fabricated, one for the top and other for the bottom containing the array of microwells. Masters for soft lithography were generated from 4-in silicon test wafers (University Wafer) coated with SU-8 2005 (MicroChem) photoresist as described elsewhere [21]. The wafer master for the bottom of the device contained five arrays of cylindrical pillars (diameter 50 micron; height 50 micron). The wafer was then fluorosilanized as described above. To avoid repeated use of the silicon wafer, we fabricated secondary masters in PDMS as follows. A total of 40 g of degassed PDMS 10:1 (base:curing agent) was poured and cured on the wafer, and then peeled off and cut into a rectangular slab. The surface containing an array of microwells was oxidized in plasma chamber (Harrick Plasma) for approximately 2 min and immediately fluorosilanized. Using this microwell-containing slab as a master, approximately 10 g of degassed PDMS was cured on it and peeled off. This new PDMS slab containing array of pillars is an exact replica of the silicon wafer, and is fluorosilanized and served as a secondary master for soft lithography for microfabrication of the bottom part of the microfluidic flowcell device. Approximately 2 g of degassed PDMS 9:1 (base: curing agent) was poured on a plasma cleaned glass slide and the secondary master with pillar array was placed gently with pillars immersed into the liquid PDMS. The slide, PDMS and master (on top) was degassed for approximately 5 min and then cured hard at 90 °C for 2 h. After curing, the master is peeled off and a thin layer of PDMS is bonded to the glass slide with five lane arrays of microwells.

A second silicon wafer master was constructed containing five longitudinal ridges (with a height of 100 microns) with rounded ends on which approximately 30 g of degassed PDMS 15:1 (base : curing agent) was poured and allowed to cure partially at 60 °C for 90 min. The partially cured PDMS was cut into a slab, holes were punched at either end of each channel, and the slab was placed gently on the top of the glass slide containing the microwell array in such a way that the longitudinal grooves were aligned over each of the five microwell arrays. The slide assembly was then incubated at 90 °C overnight to form a single monolithic PDMS structure as shown in Fig. 4a.

### Synthesis of uniquely barcoded beads for mRNA capture

N-succinimide-coated Sepharose beads with a mean diameter of approximately 30 μm were obtained from GE Healthcare in isopropanol. The beads were washed three times with water by configuration and re-suspended in a reaction mixture with a final concentration of 100 mM sodium borate (pH 8.5) and approximately 0.8 mg/mL streptavidin (streptavidin from New England BioLabs was spiked with approximately 2 % AlexaFluor 647-labeled streptavidin from Life Technologies). The reaction was incubated at room temperature for one hour on a rotisserie to allow the streptavidin to covalently attach to the beads. The beads were then washed five times in Wash Buffer and incubated in Wash Buffer for 30 min before using to completely quench any remaining reactive groups on the beads.

We annealed a dual-biotinylated oligonucleotide containing both the T7 promoter sequence and a partial Illumina adapter sequence (Additional file 1: Table S1) to each of 96 oligonucleotides (Additional file 1: Table S2) that are complementary to the partial Illumina adapter sequence on the 3′-end and contain a unique barcode and universal anchor sequence on the 5′-end (Fig. 3). The dual-biotinylated oligonucleotide was annealed at a final concentration of 2 μM in the presence of a four-fold molar excess of the barcoded oligonucleotide in a 96-well plate by stepwise cooling from 85 °C to 30 °C over 30 min. A DNA polymerase master mix was then added to each well such that the final concentration of the reaction components was 1× NEB Buffer 2 (New England BioLabs), 0.25 U/μL Klenow Fragment (exo-) (New England BioLabs), and 0.5 mM dNTPs. The reaction was incubated in each well at 37 °C for 30 min before heat inactivating the polymerase at 75 °C for 20 min.

An equal volume of beads was then added to each reaction mixture so that the extended, dual-biotinylated oligonucleotide could conjugate to the streptavidin coated beads at a final density of approximately 1 billion oligonucleotide primers per bead. The conjugation reaction was incubated at room temperature overnight on a rotisserie and quenched with biotin at a final concentration of 2 mM and sodium hydroxide at a final concentration of 125 mM to melt the template strand off of the beads. The beads were then pooled and washed five times in 125 mM sodium hydroxide supplemented with 0.1 mM biotin and then washed an additional three times with Wash Buffer and 0.1 mM biotin. The beads were then re-suspended in Hybridization Buffer (20 mM Tris pH 8.0, 1 M NaCl, 0.1 % Tween-20) supplemented with 0.1 mM biotin.

The pooled beads were split into ten reactions to which one of 10 partially complementary oligonucleotides (Additional file 1: Table S3) each containing a specific second barcode was added at a final concentration of 5 μM. The second barcode-containing oligonucleotides were allowed to hybridize to the beads at room temperature overnight on a rotisserie. The beads were then washed five times in Wash Buffer supplemented with 0.1 mM biotin and then re-suspended in a reaction mixture with final concentrations of 0.5 mM dNTPs, 1× NEB Buffer 2 (New England BioLabs), and 0.1 mM biotin. We included biotin in the wash and storage buffers in order to saturate any remaining streptavidin sites on the beads so that, in the even that a barcoded capture primer dissociates form a beads, it cannot re-associate with a different bead. The reactions were cooled to 16 °C on a thermocycler and Klenow Fragment (exo-) (New England BioLabs) was added at a final concentration of 0.25 U/μL. The reaction was incubated for 1 h at 16 °C with mixing every 10 min with a pipette followed by heat inactivation at 75 °C for 20 min.

The 10 reaction mixtures were then quenched and the hybridized strand was denatured by addition of sodium hydroxide to a final concentration of 125 mM. The reaction mixtures were then washed five times in 125 mM sodium hydroxide with 0.1 mM biotin, pooled, and then further washed three times with Wash Buffer supplemented with 0.1 mM biotin.

### Procedure for single-cell RNA-Seq experiment 1

Prior to the experiment, each lane of the device was flushed with 0.1 % Tween-20 solution and incubated for several hours to hydrate the microwells, which were subsequently washed with 2 mL of phosphate-buffered saline (PBS). Cell suspensions were counted using Countess automated cell counter (Life Technologies). A suspension of cells in PBS mixed with Calcein AM (live stain) dye was flowed in to each lane and incubated for approximately 5 min, so that the cells load in to the microwells under gravity. After thoroughly washing out the excess cells with PBS, a suspension of barcoded capture beads that had been pre-counted by microscopy was introduced in PBS and allowed to load under gravity for 5 min. We typically introduce approximately 3,000 cells to each lane of our device. It may be possible in future studies to load fewer cells and simply incubate the cell suspension for longer in order to maximize capture efficiency. We also note that only 25 % of the lower surface of each channel contains a microwell array, and so by expanding this area, we could significantly increase the number of cells captured without incurring increased reagent costs for on-chip library generation (as long as we concomitantly increased the size of our barcode pool). Excess beads were washed out thoroughly with PBS and the flow cell was incubated on ice. A total of 20 μL 0.08 % TritonX-100 (Sigma) supplemented with SUPERaseIN in PBS was flowed under ice-cold conditions immediately followed by fluorinert oil (Sigma) to seal the device. After two cycles of freeze-thaw at −80 °C to enhance cell lysis, the device was incubated at room temperature for 60 min for mRNA capture (Fig. 2a).

Two of the lanes contained pure U87 and MCF10a cells, respectively, and other lanes were loaded with a mixture of both the cell types. All lanes were imaged twice, first with blue laser (λ_ex_ = 473 nm, Dragon Lasers) for imaging the cells and secondly with a red laser (λ_ex_ = 637 nm, Obis, Coherent) for imaging the beads labeled with AlexaFluor 647 tagged streptavidin. We used the two-color images to determine number of bead-cell pairs in the array. After 1 h of incubation for mRNA capture, all the lanes were unsealed by rapid washing of the oil with 20 mM Tris, containing 1 % TritonX-100 and SUPERaseIN, followed by Wash Buffer supplemented with SUPERaseIN. After this point the microwells stay open and subsequent enzymatic steps occur simultaneously in separate lanes of the open device.

The single-cell library preparation protocol is adopted from the recently reported CEL-Seq protocol [9] with few modifications as described below. The mRNA captured on the beads was reverse transcribed using ProtoScript II Reverse Transcriptase (New England Biolabs) for 2 h at 42 °C in 1× ProtoScript Reverse Transcriptase buffer, supplemented with 10 mM DTT, 0.5 mM dNTPs, 0.1 % Tween-20 and SUPERaseIN. The reaction mixture was washed out with Wash Buffer. The second strand synthesis was carried out using reagents from the MessageAmp II aRNA amplification kit (Ambion), where a mixture of DNA polymerase and RNaseH in second strand buffer was used along with dNTPs by incubating the device at 16 °C for 2 h. After flushing out the second strand reaction mixture with Wash Buffer, an *in vitro* transcription mixture from the MessageAmp II kit containing four nucleotides and T7 RNA polymerase enzyme mix in T7 buffer was introduced to all lanes and incubated for 13 h at 37 °C (Fig. 4b). The reaction linearly amplified our cDNA, eluting pools of barcoded aRNA into the flow channels of the device which was then removed from each lane using a pipette and purified separately using RNA Clean & Concentrator columns (Zymo) and eluted into five separate tubes. The aRNA from the five lanes was reverse transcribed separately using random hexamers tagged with five different barcodes and 8-base UMIs to differentiate cDNA for all five lanes and part of an Illumina sequencing adapter. The aRNA along with the hexamer primers was heated to 70 °C for 2 min and immediately placed on ice for 5 min. The reverse transcription mix containing PrimeScript Reverse Transcriptase (Clontech-Takara), 0.5 mM dNTPs, 10 mM DTT, 1× PrimeScript buffer supplemented with SUPERaseIN was added and incubated at 25 °C for 10 min followed by 2 h incubation at 42 °C. The RNA-cDNA hybrid product was purified twice using 0.65× ratio of Agencourt Ampure beads (Beckman Coulter) and the purified cDNA from all the lanes were pooled together for PCR. Phusion High Fidelity DNA polymerase (New England Biolabs) was used for amplifying the cDNA using RP1 and RPI Illumina primers in 1× PhusionHF buffer supplemented with dNTPs. The PCR product was purified on a 1.5 % agarose gel which was stained with SybrGold (Life Technologies) before being cut between 400–800 bp. The library was extracted from the gel using Gel Extraction kit (Qiagen), and further purified and concentrated using a 0.65× ratio of the AMpure beads (Beckman Coulter). The final library was quantified usinga Qubit (Life Technologies) and Bioanalyzer (Agilent) and sequenced on NextSeq 500 desktop sequencer (Illumina). We obtained approximately 240 million paired-end reads with a 26-base first read and a 66-base second read.

### Procedure for single-cell RNA-Seq experiment 2

Experiment 2 was identical to Experiment 1 with a few exceptions. First, the two cell types under study were U87 human glioma cells and WI-38 human fibroblast cells (a diploid, limited-passage, non-cancer cell line). Second, reagents from the HiScribe *In Vitro* Transcription kit (New England BioLabs) were substituted for the MessageAmp II kit for the IVT portion of the protocol. Third, some of the oligonucleotides used were different from in Experiment 1 and are tabulated (Additional file 1: Tables S4–6). Finally, we obtained a sufficiently pure library that gel purification was unwarranted and library purification using AMpure beads was sufficient. We obtained approximately 130 million paired-end reads with a 26-base first reads and a 60-base second read on a NextSeq 500 desktop sequencer.

### Analysis of single-cell RNA-Seq data

Read 1 of our single-cell RNA-Seq data contains a cell-identifying barcode sequence followed by poly(dT), and read 2 contains a 8-base UMI followed by a 6-base lane-identifying barcode and a transcript sequence. We first demultiplex the reads based on the lane-identifying barcode while recording the corresponding UMI using a custom Python script. We then map the remainder of read 2 to the human genome and transcriptome (hg19, Ensembl annotation from Illumina iGenomes) using the STAR aligner [40]. Mapped reads for each lane are then demultiplexed based on the cell-identifying barcodes in read 1 and assigned to a gene using HTSeq [41]. Both the lane- and cell-identifying barcodes were allowed to have a single-base mismatch during demultiplexing.

We collected the set of reads that uniquely mapped to the transcriptome and assigned an address comprised of its cell-identifying barcode, gene, UMI, and mapping position. In addition, we kept reads that mapped to both the genome and transcriptome, but that mapped to only one position on the transcriptome and mapped to that position with the appropriate strand-specificity. We then filtered the reads to identify unique molecules. Reads with identical addresses were collapsed to a single molecule. In addition, reads with identical cell-identifying barcodes, genes, mapping positions, and with UMIs having a Hamming distance less than or equal to two were collapsed to a single molecule. Because the mapping positions produced by STAR do not necessarily correspond to the beginning of a read, we further considered reads to originate from identical molecules if they had identical genes, cell-identifying barcodes, UMIs with a Hamming distance less than or equal to two, and a mapping position within six bases. Finally, we removed all reads considered identical molecules by the above definition (UMIs with a Hamming distance less than or equal to two and mapping position within six bases) but that also occurred with different cell-identifying barcodes within the same lane. This conservative approach likely underestimates of the true number of molecules associated with each cell and gene and results in some loss of gene detection. However, it also removes molecules that may become spuriously associated with the incorrect cell via PCR recombination, as observed and similarly filtered in previous studies that used very similar library construction protocols [12].

To identify barcodes that correspond to actual individual cells in our device in Experiment 1, we filtered the observed cell-identifying barcodes by progressively downsampling the corresponding gene profiles to the same number of total reads and assessing the number of unique molecules detected from each cell-identifying barcode. After excluding cell-identifying barcodes having zero associated molecules, we found the distribution of associated unique molecules to be bimodal, with one small subpopulation having nearly as many unique molecules as reads at low read totals (Additional file 1: Figure S1a, b). We found the size of this subpopulation to be in excellent agreement with our device imaging data (Additional file 1: Figures S1c, S2). We took these 598 profiles to represent the actual individual cells captured in our device with a barcoded bead. We used the same approach to assess the cell-identifying barcodes in Experiment 2.

We kept the 396 single-cell profiles with the highest coverage in our dataset (all five lanes represented). We compared the U87 and MCF10a single-cell profiles to bulk RNA-Seq profiles of U87 and MCF10a cells. We prepared a bulk RNA-Seq library from approximately 10^7^ U87 cells using the TruSeq RNA-Seq library preparation kit (Illumina) and sequenced the library to a depth of approximately 30 M, 100-base single-end reads on an Illumina HiSeq 2500. We obtained a publically available bulk RNA-Seq profile of MCF10a cells from the Gene Expression Omnibus (entry GSE45258). Reads were mapped to the transcriptome as described above and expression values (FPKM) were computed using Cufflinks [42]. Pearson correlation coefficients between single-cell and bulk profiles were computed between log-transformed single-cell expression profiles (unique molecules per million reads plus one) and log-transformed bulk values (FPKM plus one). We generated single-cell median profiles from different numbers of randomly selected single-cell profiles and repeated this random sampling 10 times without replacement for each data point in Fig. 6a and b. For each Pearson correlation calculation, only genes with log-transformed single-cell median or bulk values greater than 0.5 were included.

Differential expression analysis was conducted by comparing each detected gene in the two cell type-exclusive lanes using Wilcoxon’s rank-sum test. Genes with *P* <0.05 were used for clustering analysis. Regardless of differential expression we used +/−(1-p) (which is positive for expression biased in one cell type and negative for expression biased towards a second cell type) for each gene as input to iPAGE, a mutual information-based algorithm that can associate gene ontologies with genes based on an assigned numerical value [35]. We then generated a matrix of pairwise Spearman correlation coefficients based on unique molecules detected across 396 single-cell profiles in Experiment 1 (247 profiles in Experiment 2) using only the differentially expressed genes. We then clustered the data with the MATLAB implementation t-SNE using the correlation matrix as input. We color-coated the single-cell profiles in the t-SNE clusters using a simple classifier score given by the log-ratio of the number of cell type-specific genes for each of the two cell types in a given cell with an above-average rank in expression level (Fig. 7c, Additional file 1: Figure S4b).

## Additional file

Additional file 1:
**Figures S1–4 including analysis of barcodes, image of RNA capture in an unsealed microwell device, clustering analysis of Experiment 2, Tables S1–6 containing oligonucleotide sequences, and Table S7 detailing reagents costs.**

## Abbreviations

aRNA: Amplified RNA
ISA: Illumina sequencing adapter
LNA: Locked nucleic acid
PDMS: Polydimethylsiloxane
qRT-PCR: Quantitative real-time polymerase chain reaction
RNA-FISH: RNA fluorescence *in situ* hybridization
RNA-Seq: RNA sequencing
TPS: T7 promoter sequence
tSNE: t-stochastic neighborhood embedding
UMI: Unique molecular identifier
