# Supplementary material for: Scalable microfluidics for single-cell RNA printing and sequencing
Source: Genome Biol. 2015 Jun 6;16(1):120. doi: 10.1186/s13059-015-0684-3 (PMC4487847; doi:10.1186/s13059-015-0684-3)
Supplement: Additional file 1: — Figures S1–4 including analysis of barcodes, image of RNA capture in an unsealed microwell device, clustering analysis of Experiment 2, Tables S1–6 containing oligonucleotide sequences, and Table S7 detailing reagents costs. [file 13059_2015_684_MOESM1_ESM.pdf]

Supplementary Figures

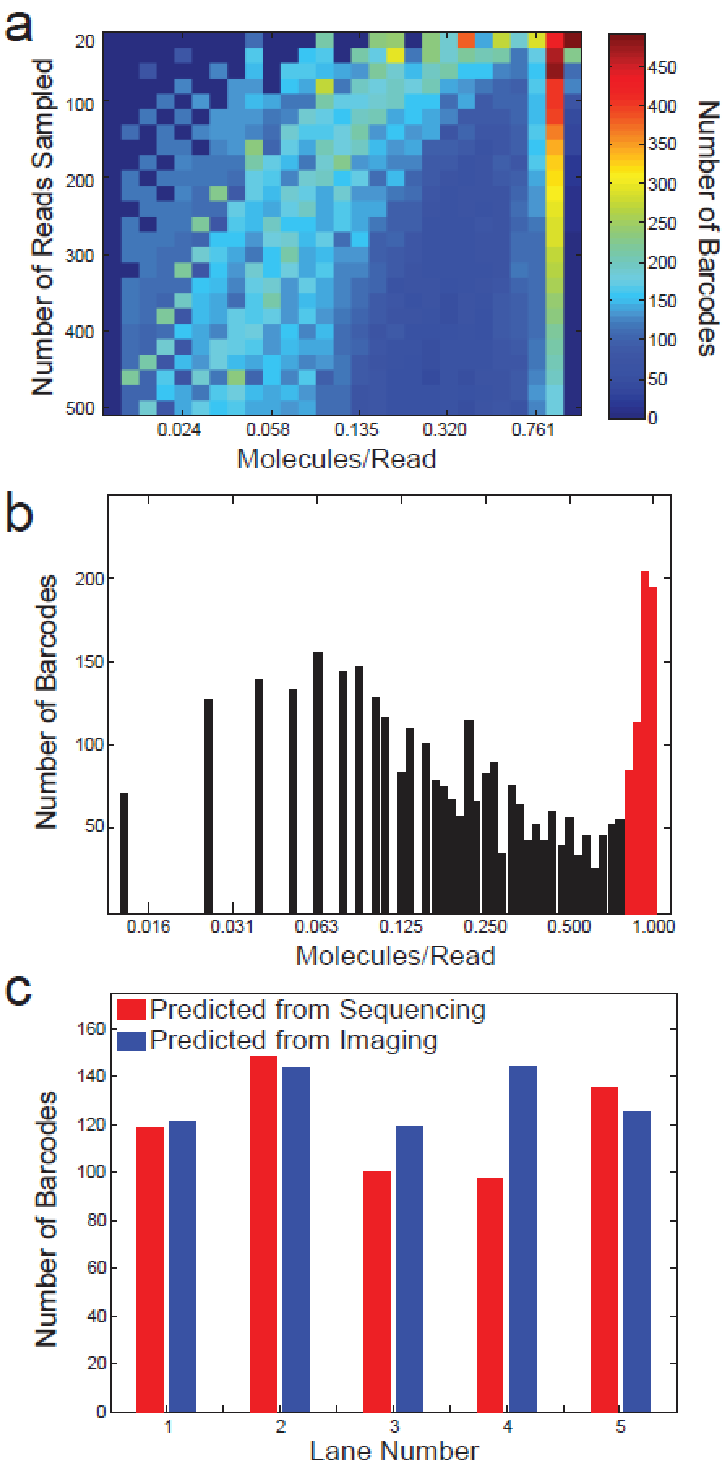

Supplementary Figure 1. Analysis of cell-identifying barcodes. **a)** Expression profiles for all detected cell-identifying barcodes were downsampled to between 20 and 500 unique molecules in regular increments (y-axis of heatmap) and the distribution of number of molecules per read (x-axis) is

histogrammed across the heatmap. The series of histograms reveals two clear populations of barcodes. The barcodes in the population on the left shift continuously to the left and are associated with relatively few molecules per read compared to the population on the far right which have closer to one molecule per read. We take this small subpopulation of high-coverage barcodes to indicate the actual single cell RNA samples captured in our device. **b)** A single histogram from the heatmap in **a)** where the profiles have been downsampled to 100 reads per barcode. The high-coverage subpopulation is highlighted in red. **c)** Comparison between the number of unique cell-identifying barcodes predicted from the high-coverage subpopulation in **a)** and **b)** and the number of unique cell-identifying barcodes expected from imaging bead-cell pairs in the microwells in our device by fluorescence microscopy (**Fig. 4c**). Note that the number of cell-identifying barcodes in each lane was determined based on a single threshold for the whole data set with barcodes from all lanes pooled together (we didn't need to choose a different threshold for each lane to get these results).

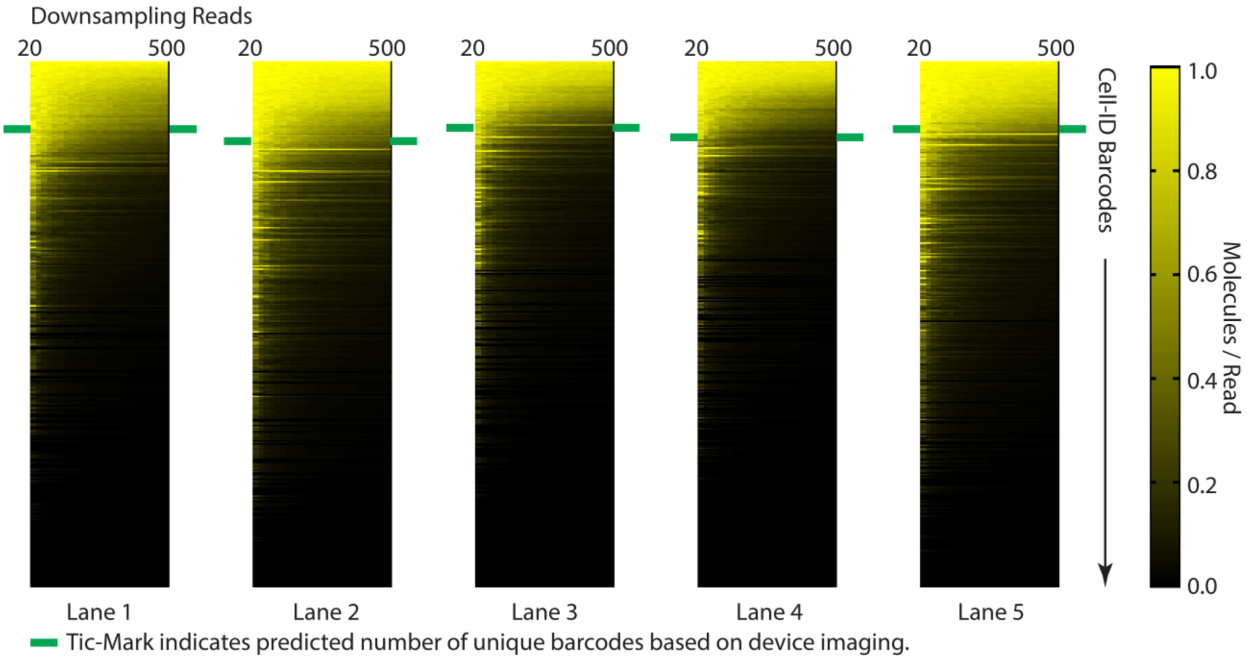

Supplementary Figure 2. Heatmap display of downsampling analysis in **Supplementary Fig. 1a** on a lane-by-lane and barcode-by-barcode basis where the downsampled distributions are shown for all 960 cell-identifying barcodes in each lane after ordering the barcodes by total coverage. Green tic-marks indicate the predicted number of cell-identifying barcodes based on imaging cell-bead pairs in the device.

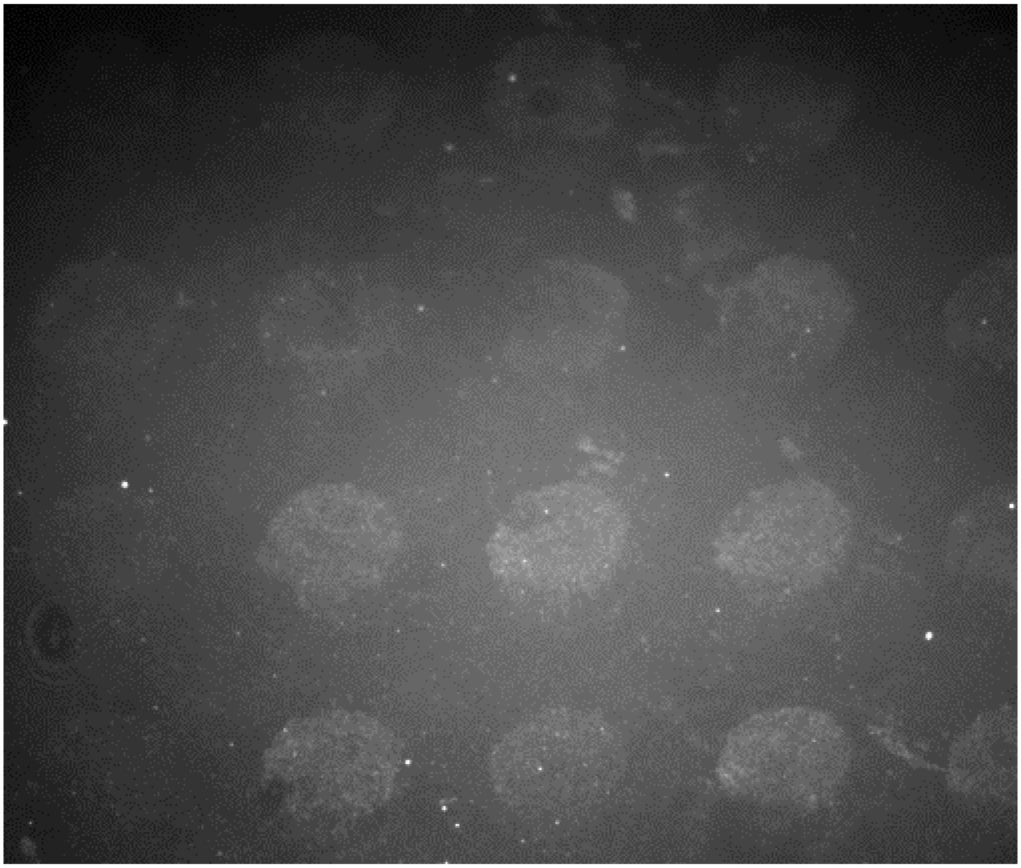

Supplementary Figure 3. Fluorescence image of a glass coverslip after single cell RNA capture, reverse transcription, and Sytox Orange staining using the scheme in **Fig. 1a** with an improperly or partially sealed microwell array demonstrating the rapid escape and uniform distribution of RNA by diffusion from individual cells into every printing site.

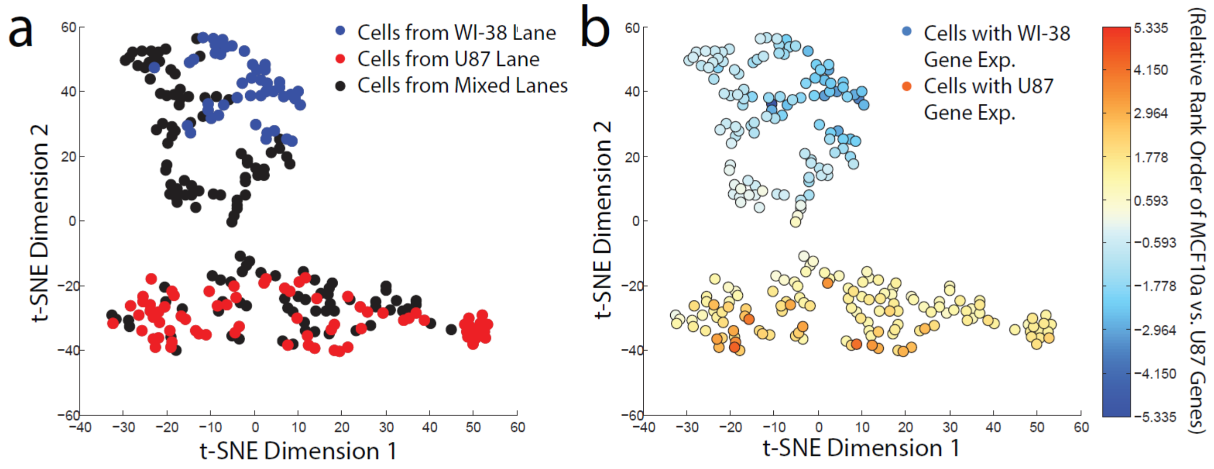

Supplementary Figure 4. Cell type separation from single cell RNA-Seq Experiment 2. **a)** t-SNE clustering of 247 single cell profiles based on differentially expressed genes color-coated by the lane-of-origin of each profile. Two clear spatial clusters form and each is exclusively associated with a specific cell type-exclusive lane. **b)** The same t-SNE clustering shown in **a)** but color-coated with a score indicating expression of the U87-specific genes vs. the WI-38-specific genes. The score is based on the relative rank-ordering of WI-38 and U87-specific genes in each cell (see Methods).

Supplementary Tables

Table 1. List of key oligonucleotides used for barcoding and library preparation (not including the long list of cell-identifying barcodes that appear in the subsequent tables) for Experiment 1.

Oligonucleotide Name	Oligonucleotide Sequence
Bead Capture Oligo (5'-dual biotinylated)	AGGTAAGGTAATACGACTCACTATAGGGGTTCAGAGTTCTACAGTCCGACGATC
RT1 (Reverse Transcription Primer for Lane 1)	GCCTTGGCACCCGAGAATTCCANNNNNNNNCGTGATNNNNNN
RT2 (Reverse Transcription Primer for Lane 2)	GCCTTGGCACCCGAGAATTCCANNNNNNNNACATCGNNNNNN
RT3 (Reverse Transcription Primer for Lane 3)	GCCTTGGCACCCGAGAATTCCANNNNNNNNGCCTAANNNNNN
RT4 (Reverse Transcription Primer for Lane 4)	GCCTTGGCACCCGAGAATTCCANNNNNNNNTGGTCANNNNNN
RT5 (Reverse Transcription Primer for Lane 5)	GCCTTGGCACCCGAGAATTCCANNNNNNNNCACTGTNNNNNN
RP1 (PCR Primer 1)	AATGATACGGCGACCACCGAGATCTACACGTTCAGAGTTCTACAGTCCGA
RPI1 (PCR Primer 2)	CAAGCAGAAGACGGCATACGAGATCGTGATGTGACTGGAGTTCCTTGGCACCCGAGAATTCCA

Table 2. Oligonucleotide sequences used to generate the first set of barcoded beads (FBC) for combinatorial synthesis in Experiment 1.

Oligonucleotide Name	Oligonucleotide Sequence
FBC_Oligo1	CAGGTCAACCAGAGAGATCGTCGGACTGTAGAACTCTGAAC
FBC_Oligo2	CAGGTCAAAGTACGCGATCGTCGGACTGTAGAACTCTGAAC
FBC_Oligo3	CAGGTCGTTTGGCATGATCGTCGGACTGTAGAACTCTGAAC
FBC_Oligo4	CAGGTCAAGTGAGGTGATCGTCGGACTGTAGAACTCTGAAC
FBC_Oligo5	CAGGTCACGTTAGCTGATCGTCGGACTGTAGAACTCTGAAC
FBC_Oligo6	CAGGTCGTGCTAGAGGATCGTCGGACTGTAGAACTCTGAAC
FBC_Oligo7	CAGGTCGTCCTGTGTGATCGTCGGACTGTAGAACTCTGAAC
FBC_Oligo8	CAGGTCTCTACGGCAGATCGTCGGACTGTAGAACTCTGAAC
FBC_Oligo9	CAGGTCACAGGGCTTGATCGTCGGACTGTAGAACTCTGAAC
FBC_Oligo10	CAGGTCGTGCGTTATGATCGTCGGACTGTAGAACTCTGAAC
FBC_Oligo11	CAGGTCGGGTAAGTAGATCGTCGGACTGTAGAACTCTGAAC
FBC_Oligo12	CAGGTCTCCCTTAGGGATCGTCGGACTGTAGAACTCTGAAC
FBC_Oligo13	CAGGTCCAAGTTGGTGATCGTCGGACTGTAGAACTCTGAAC
FBC_Oligo14	CAGGTCTTCTCACTCGATCGTCGGACTGTAGAACTCTGAAC
FBC_Oligo15	CAGGTCTCCCACTCTGATCGTCGGACTGTAGAACTCTGAAC
FBC_Oligo16	CAGGTCCGGTATACCGATCGTCGGACTGTAGAACTCTGAAC
FBC_Oligo17	CAGGTCAGGCATGTGGATCGTCGGACTGTAGAACTCTGAAC
FBC_Oligo18	CAGGTCCCCAGATTGGATCGTCGGACTGTAGAACTCTGAAC
FBC_Oligo19	CAGGTCTTCCCTTGAGATCGTCGGACTGTAGAACTCTGAAC
FBC_Oligo20	CAGGTCGTTGTACGAGATCGTCGGACTGTAGAACTCTGAAC
FBC_Oligo21	CAGGTCTGCTTGCAGGATCGTCGGACTGTAGAACTCTGAAC
FBC_Oligo22	CAGGTCGGCCTCATTGATCGTCGGACTGTAGAACTCTGAAC
FBC_Oligo23	CAGGTCAACAGCCTAGATCGTCGGACTGTAGAACTCTGAAC

FBC_Oligo24	CAGGTCGATGCAATGGATCGTCGGACTGTAGAACTCTGAAC
FBC_Oligo25	CAGGTCGAAGGAACGGATCGTCGGACTGTAGAACTCTGAAC
FBC_Oligo26	CAGGTCCAGCCACTTGATCGTCGGACTGTAGAACTCTGAAC
FBC_Oligo27	CAGGTCCTCTGCTTCGATCGTCGGACTGTAGAACTCTGAAC
FBC_Oligo28	CAGGTCGGCTTATGAGATCGTCGGACTGTAGAACTCTGAAC
FBC_Oligo29	CAGGTCCTAGTCCTCGATCGTCGGACTGTAGAACTCTGAAC
FBC_Oligo30	CAGGTCCTAGAGGAGGATCGTCGGACTGTAGAACTCTGAAC
FBC_Oligo31	CAGGTCAGCTTTACCGATCGTCGGACTGTAGAACTCTGAAC
FBC_Oligo32	CAGGTCGTCCATGAAGATCGTCGGACTGTAGAACTCTGAAC
FBC_Oligo33	CAGGTCCTCGAACCTGATCGTCGGACTGTAGAACTCTGAAC
FBC_Oligo34	CAGGTCCATTGTACGGATCGTCGGACTGTAGAACTCTGAAC
FBC_Oligo35	CAGGTCTTGAACGCTGATCGTCGGACTGTAGAACTCTGAAC
FBC_Oligo36	CAGGTCTACGTCATGGATCGTCGGACTGTAGAACTCTGAAC
FBC_Oligo37	CAGGTCAAGCCGTTAGATCGTCGGACTGTAGAACTCTGAAC
FBC_Oligo38	CAGGTCCGGACGTATGATCGTCGGACTGTAGAACTCTGAAC
FBC_Oligo39	CAGGTCTCGTTACCGGATCGTCGGACTGTAGAACTCTGAAC
FBC_Oligo40	CAGGTCATCCCCCATGATCGTCGGACTGTAGAACTCTGAAC
FBC_Oligo41	CAGGTCCAGACGATTGATCGTCGGACTGTAGAACTCTGAAC
FBC_Oligo42	CAGGTCATCGATCCCGATCGTCGGACTGTAGAACTCTGAAC
FBC_Oligo43	CAGGTCCCTGAGGATGATCGTCGGACTGTAGAACTCTGAAC
FBC_Oligo44	CAGGTCAGCTCTTTGGATCGTCGGACTGTAGAACTCTGAAC
FBC_Oligo45	CAGGTCGGAATACGGGATCGTCGGACTGTAGAACTCTGAAC
FBC_Oligo46	CAGGTCCTATCCTGGGATCGTCGGACTGTAGAACTCTGAAC
FBC_Oligo47	CAGGTCGGTTGTAGTGATCGTCGGACTGTAGAACTCTGAAC
FBC_Oligo48	CAGGTCGAACGTAGCGATCGTCGGACTGTAGAACTCTGAAC
FBC_Oligo49	CAGGTCGTCTATCGGATCGTCGGACTGTAGAACTCTGAAC
FBC_Oligo50	CAGGTCTACGAGTGGATCGTCGGACTGTAGAACTCTGAAC
FBC_Oligo51	CAGGTCTCATGTCGGATCGTCGGACTGTAGAACTCTGAAC
FBC_Oligo52	CAGGTCAAACACCCGATCGTCGGACTGTAGAACTCTGAAC
FBC_Oligo53	CAGGTCACTAGTCCGATCGTCGGACTGTAGAACTCTGAAC
FBC_Oligo54	CAGGTCCGAGGAATGATCGTCGGACTGTAGAACTCTGAAC
FBC_Oligo55	CAGGTCACAATGGCGATCGTCGGACTGTAGAACTCTGAAC
FBC_Oligo56	CAGGTCTAGGTCTCGATCGTCGGACTGTAGAACTCTGAAC
FBC_Oligo57	CAGGTCTCTGTGAGGATCGTCGGACTGTAGAACTCTGAAC
FBC_Oligo58	CAGGTCGGGATTGAGATCGTCGGACTGTAGAACTCTGAAC
FBC_Oligo59	CAGGTCAACTCTGGGATCGTCGGACTGTAGAACTCTGAAC
FBC_Oligo60	CAGGTCAAACGCGTGATCGTCGGACTGTAGAACTCTGAAC
FBC_Oligo61	CAGGTCTCCTACGAGATCGTCGGACTGTAGAACTCTGAAC
FBC_Oligo62	CAGGTCTAGCAGGTGATCGTCGGACTGTAGAACTCTGAAC
FBC_Oligo63	CAGGTCCCTGCATTGATCGTCGGACTGTAGAACTCTGAAC
FBC_Oligo64	CAGGTCGTGATGCAGATCGTCGGACTGTAGAACTCTGAAC
FBC_Oligo65	CAGGTCCGATTCAGGATCGTCGGACTGTAGAACTCTGAAC
FBC_Oligo66	CAGGTCAGGATGACGATCGTCGGACTGTAGAACTCTGAAC
FBC_Oligo67	CAGGTCAGGCCATAGATCGTCGGACTGTAGAACTCTGAAC
FBC_Oligo68	CAGGTCGCTTGCTTGATCGTCGGACTGTAGAACTCTGAAC
FBC_Oligo69	CAGGTCTCCCAAGTGATCGTCGGACTGTAGAACTCTGAAC
FBC_Oligo70	CAGGTCTCAAGGCAGATCGTCGGACTGTAGAACTCTGAAC
FBC_Oligo71	CAGGTCACGAGGTAGATCGTCGGACTGTAGAACTCTGAAC
FBC_Oligo72	CAGGTCGGAACGAAGATCGTCGGACTGTAGAACTCTGAAC

FBC_Oligo73	CAGGTCAATCCCAGGATCGTCGGACTGTAGAACTCTGAAC
FBC_Oligo74	CAGGTCCGATAAGGGATCGTCGGACTGTAGAACTCTGAAC
FBC_Oligo75	CAGGTCTATCGCGAGATCGTCGGACTGTAGAACTCTGAAC
FBC_Oligo76	CAGGTCCGCATAACGATCGTCGGACTGTAGAACTCTGAAC
FBC_Oligo77	CAGGTCGTGCAGTTGATCGTCGGACTGTAGAACTCTGAAC
FBC_Oligo78	CAGGTCAGAACGCTGATCGTCGGACTGTAGAACTCTGAAC
FBC_Oligo79	CAGGTCTAGAGGTCGATCGTCGGACTGTAGAACTCTGAAC
FBC_Oligo80	CAGGTCCTGTGATGGATCGTCGGACTGTAGAACTCTGAAC
FBC_Oligo81	CAGGTCTAGAGCCAGATCGTCGGACTGTAGAACTCTGAAC
FBC_Oligo82	CAGGTCCTTGATGCGATCGTCGGACTGTAGAACTCTGAAC
FBC_Oligo83	CAGGTCTTCGTGTCGATCGTCGGACTGTAGAACTCTGAAC
FBC_Oligo84	CAGGTCTATCTGCGGATCGTCGGACTGTAGAACTCTGAAC
FBC_Oligo85	CAGGTCTGGTAGGAGATCGTCGGACTGTAGAACTCTGAAC
FBC_Oligo86	CAGGTCCCTAGACAGATCGTCGGACTGTAGAACTCTGAAC
FBC_Oligo87	CAGGTCAGTCAACGGATCGTCGGACTGTAGAACTCTGAAC
FBC_Oligo88	CAGGTCAAGGGTGAGATCGTCGGACTGTAGAACTCTGAAC
FBC_Oligo89	CAGGTCCTTCACACGATCGTCGGACTGTAGAACTCTGAAC
FBC_Oligo90	CAGGTCAGGTTGCTGATCGTCGGACTGTAGAACTCTGAAC
FBC_Oligo91	CAGGTCACCCGAAAGATCGTCGGACTGTAGAACTCTGAAC
FBC_Oligo92	CAGGTCGAAAAGGGGATCGTCGGACTGTAGAACTCTGAAC
FBC_Oligo93	CAGGTCACTTCCCAGATCGTCGGACTGTAGAACTCTGAAC
FBC_Oligo94	CAGGTCTGCTGCATGATCGTCGGACTGTAGAACTCTGAAC
FBC_Oligo95	CAGGTCATTCCTGGGATCGTCGGACTGTAGAACTCTGAAC
FBC_Oligo96	CAGGTCCAGAACTCGATCGTCGGACTGTAGAACTCTGAAC

Table 3. Oligonucleotide sequences used to generate the second set of barcoded beads (SBC) for combinatorial synthesis in Experiment 1.

Oligonucleotide Name	Oligonucleotide Sequence
SBC_Oligo1	AAAAAAAAAAAAAAAAAAAAAAAAAGGTGATACAGGTCAAAAAAAAAGATCG TCGGACTGTAGAACTC
SBC_Oligo2	AAAAAAAAAAAAAAAAAAAAAAAAATGAATGCCAGGTCAAAAAAAAAGATCG TCGGACTGTAGAACTC
SBC_Oligo3	AAAAAAAAAAAAAAAAAAAAAAAAATGCCAAACAGGTCAAAAAAAAAGATCG TCGGACTGTAGAACTC
SBC_Oligo4	AAAAAAAAAAAAAAAAAAAAAAAAAACAGAAGCAGGTCAAAAAAAAAGATCG TCGGACTGTAGAACTC
SBC_Oligo5	AAAAAAAAAAAAAAAAAAAAAAAAACACTGGACAGGTCAAAAAAAAAGATCG TCGGACTGTAGAACTC
SBC_Oligo6	AAAAAAAAAAAAAAAAAAAAAAAAACGATGATCAGGTCAAAAAAAAAGATCG TCGGACTGTAGAACTC
SBC_Oligo7	AAAAAAAAAAAAAAAAAAAAAAAAAGTGTCCACAGGTCAAAAAAAAAGATCG TCGGACTGTAGAACTC
SBC_Oligo8	AAAAAAAAAAAAAAAAAAAAAAAAATCCTCTTCAGGTCAAAAAAAAAGATCG TCGGACTGTAGAACTC
SBC_Oligo9	AAAAAAAAAAAAAAAAAAAAAAAAAGTGCAGTCAGGTCAAAAAAAAAGATCG TCGGACTGTAGAACTC
SBC_Oligo10	AAAAAAAAAAAAAAAAAAAAAAAAAAGGTAGACAGGTCAAAAAAAAAGATCG TCGGACTGTAGAACTC

Table 4. List of key oligonucleotides used for barcoding and library preparation (not including the long list of cell-identifying barcodes that appear in the subsequent tables) for Experiment 2.

Oligonucleotide Name	Oligonucleotide Sequence
Bead Capture Oligo (5'-dual biotinylated)	AGGTAAGGTAATACGACTCACTATAGGGGTTCAGAGTTCTACAGTCCGACGATC
RT1 (Reverse Transcription Primer for Lane 1)	GCCTTGGCACCCGAGAATTCCANNNNNNNNCGTCATNNNNNN
RT2 (Reverse Transcription Primer for Lane 2)	GCCTTGGCACCCGAGAATTCCANNNNNNNNTACCCANNNNNN
RT3 (Reverse Transcription Primer for Lane 3)	GCCTTGGCACCCGAGAATTCCANNNNNNNNGCCATTNNNNNN
RT4 (Reverse Transcription Primer for Lane 4)	GCCTTGGCACCCGAGAATTCCANNNNNNNNGAGTACNNNNNN
RT5 (Reverse Transcription Primer for Lane 5)	GCCTTGGCACCCGAGAATTCCANNNNNNNNAGAGTCNNNNNN
RP1 (PCR Primer 1)	AATGATACGGCGACCACCGAGATCTACACGTTCAGAGTTCTACAGTCCGA
RPI2 (PCR Primer 2)	CAAGCAGAAGACGGCATACGAGATACATCGGTGACTGGAGTTCCTTGGCACCCGAGAATTCCA

Table 5. Oligonucleotide sequences used to generate the first set of barcoded beads (FBC) for combinatorial synthesis in Experiment 2.

Oligonucleotide Name	Oligonucleotide Sequence
FBC_Oligo1	CAGGTCCTGATCGATGATCGTCGGACTGTAGAACTCTGAAC
FBC_Oligo2	CAGGTCGTGTAGACAGATCGTCGGACTGTAGAACTCTGAAC
FBC_Oligo3	CAGGTCCATTGTTCCGATCGTCGGACTGTAGAACTCTGAAC
FBC_Oligo4	CAGGTCCTTGACTACGATCGTCGGACTGTAGAACTCTGAAC
FBC_Oligo5	CAGGTCACCGTTTCGGATCGTCGGACTGTAGAACTCTGAAC
FBC_Oligo6	CAGGTCAAGGACCGTGATCGTCGGACTGTAGAACTCTGAAC
FBC_Oligo7	CAGGTCTCACTATGCGATCGTCGGACTGTAGAACTCTGAAC
FBC_Oligo8	CAGGTCCTGCAATGGGATCGTCGGACTGTAGAACTCTGAAC
FBC_Oligo9	CAGGTCTGAGTCGTCGATCGTCGGACTGTAGAACTCTGAAC
FBC_Oligo10	CAGGTCCTCACACTAGATCGTCGGACTGTAGAACTCTGAAC
FBC_Oligo11	CAGGTCTTACCCCCTGATCGTCGGACTGTAGAACTCTGAAC
FBC_Oligo12	CAGGTCCCAAGTAGAGATCGTCGGACTGTAGAACTCTGAAC
FBC_Oligo13	CAGGTCATAGCGCACGATCGTCGGACTGTAGAACTCTGAAC
FBC_Oligo14	CAGGTCTGACGTACGGATCGTCGGACTGTAGAACTCTGAAC
FBC_Oligo15	CAGGTCGTAGAGTTGGATCGTCGGACTGTAGAACTCTGAAC
FBC_Oligo16	CAGGTCTTTCTGGCGGATCGTCGGACTGTAGAACTCTGAAC
FBC_Oligo17	CAGGTCGGAATGTGTGATCGTCGGACTGTAGAACTCTGAAC
FBC_Oligo18	CAGGTCCTATGGAAGGATCGTCGGACTGTAGAACTCTGAAC
FBC_Oligo19	CAGGTCAAGTCCATGGATCGTCGGACTGTAGAACTCTGAAC
FBC_Oligo20	CAGGTCAGTACTTGGGATCGTCGGACTGTAGAACTCTGAAC
FBC_Oligo21	CAGGTCACAGGACTAGATCGTCGGACTGTAGAACTCTGAAC
FBC_Oligo22	CAGGTCACCAGGTAAGATCGTCGGACTGTAGAACTCTGAAC
FBC_Oligo23	CAGGTCGCATGAACCGATCGTCGGACTGTAGAACTCTGAAC
FBC_Oligo24	CAGGTCGTTGGTGTTGATCGTCGGACTGTAGAACTCTGAAC

FBC_Oligo25	CAGGTCCCTTCAGACGATCGTCGGACTGTAGAACTCTGAAC
FBC_Oligo26	CAGGTCCCTCTTGGTGATCGTCGGACTGTAGAACTCTGAAC
FBC_Oligo27	CAGGTCGGGAAAGTTGATCGTCGGACTGTAGAACTCTGAAC
FBC_Oligo28	CAGGTCAGCCAGAGTGATCGTCGGACTGTAGAACTCTGAAC
FBC_Oligo29	CAGGTCTCGCATCTGGATCGTCGGACTGTAGAACTCTGAAC
FBC_Oligo30	CAGGTCGATACGGCAGATCGTCGGACTGTAGAACTCTGAAC
FBC_Oligo31	CAGGTCTCGGCCAAAGATCGTCGGACTGTAGAACTCTGAAC
FBC_Oligo32	CAGGTCAGATTTCGCGATCGTCGGACTGTAGAACTCTGAAC
FBC_Oligo33	CAGGTCGACCCTCAAGATCGTCGGACTGTAGAACTCTGAAC
FBC_Oligo34	CAGGTCAGTCCACTCGATCGTCGGACTGTAGAACTCTGAAC
FBC_Oligo35	CAGGTCCAAACGATCGATCGTCGGACTGTAGAACTCTGAAC
FBC_Oligo36	CAGGTCGCCTAATAGGATCGTCGGACTGTAGAACTCTGAAC
FBC_Oligo37	CAGGTCGGCTACATCGATCGTCGGACTGTAGAACTCTGAAC
FBC_Oligo38	CAGGTCTATGAGCAGGATCGTCGGACTGTAGAACTCTGAAC
FBC_Oligo39	CAGGTCGGTAGTAACGATCGTCGGACTGTAGAACTCTGAAC
FBC_Oligo40	CAGGTCCGCGTATATGATCGTCGGACTGTAGAACTCTGAAC
FBC_Oligo41	CAGGTCTACTGGAGCGATCGTCGGACTGTAGAACTCTGAAC
FBC_Oligo42	CAGGTCAGGGAATCAGATCGTCGGACTGTAGAACTCTGAAC
FBC_Oligo43	CAGGTCATCCGAGATGATCGTCGGACTGTAGAACTCTGAAC
FBC_Oligo44	CAGGTCTCCCAAGCAGATCGTCGGACTGTAGAACTCTGAAC
FBC_Oligo45	CAGGTCGAGCCGTTTGATCGTCGGACTGTAGAACTCTGAAC
FBC_Oligo46	CAGGTCTGCTCTTACGATCGTCGGACTGTAGAACTCTGAAC
FBC_Oligo47	CAGGTCACGACTACCGATCGTCGGACTGTAGAACTCTGAAC
FBC_Oligo48	CAGGTCCAAGCAGCTGATCGTCGGACTGTAGAACTCTGAAC
FBC_Oligo49	CAGGTCGTATTCGCGATCGTCGGACTGTAGAACTCTGAAC
FBC_Oligo50	CAGGTCGCTCTGAAGATCGTCGGACTGTAGAACTCTGAAC
FBC_Oligo51	CAGGTCACGTAGTGGATCGTCGGACTGTAGAACTCTGAAC
FBC_Oligo52	CAGGTCATTGGGTCGATCGTCGGACTGTAGAACTCTGAAC
FBC_Oligo53	CAGGTCAACAGCACGATCGTCGGACTGTAGAACTCTGAAC
FBC_Oligo54	CAGGTCTCAGAGACGATCGTCGGACTGTAGAACTCTGAAC
FBC_Oligo55	CAGGTCGTGTGCTAGATCGTCGGACTGTAGAACTCTGAAC
FBC_Oligo56	CAGGTCGCAGTTGAGATCGTCGGACTGTAGAACTCTGAAC
FBC_Oligo57	CAGGTCTTAACGGGGATCGTCGGACTGTAGAACTCTGAAC
FBC_Oligo58	CAGGTCGCTCGATTGATCGTCGGACTGTAGAACTCTGAAC
FBC_Oligo59	CAGGTCACACCTGTGATCGTCGGACTGTAGAACTCTGAAC
FBC_Oligo60	CAGGTCAGACGGTTGATCGTCGGACTGTAGAACTCTGAAC
FBC_Oligo61	CAGGTCGCAAACCAGATCGTCGGACTGTAGAACTCTGAAC
FBC_Oligo62	CAGGTCGAGTATGGGATCGTCGGACTGTAGAACTCTGAAC
FBC_Oligo63	CAGGTCGGTCTTTCGATCGTCGGACTGTAGAACTCTGAAC
FBC_Oligo64	CAGGTCCATCTGCTGATCGTCGGACTGTAGAACTCTGAAC
FBC_Oligo65	CAGGTCTTCGCAAGGATCGTCGGACTGTAGAACTCTGAAC
FBC_Oligo66	CAGGTCTTGTGACGGATCGTCGGACTGTAGAACTCTGAAC
FBC_Oligo67	CAGGTCTGCATGACGATCGTCGGACTGTAGAACTCTGAAC
FBC_Oligo68	CAGGTCCAACGTGAGATCGTCGGACTGTAGAACTCTGAAC
FBC_Oligo69	CAGGTCTAGGCTTCGATCGTCGGACTGTAGAACTCTGAAC
FBC_Oligo70	CAGGTCTGGTAGGAGATCGTCGGACTGTAGAACTCTGAAC
FBC_Oligo71	CAGGTCTGCAGCTTGATCGTCGGACTGTAGAACTCTGAAC
FBC_Oligo72	CAGGTCCTGTACCTGATCGTCGGACTGTAGAACTCTGAAC
FBC_Oligo73	CAGGTCCGCAATGAGATCGTCGGACTGTAGAACTCTGAAC

FBC_Oligo74	CAGGTCGATCCAAGGATCGTCGGACTGTAGAACTCTGAAC
FBC_Oligo75	CAGGTCCACTTACGGATCGTCGGACTGTAGAACTCTGAAC
FBC_Oligo76	CAGGTCAACTAGGCGATCGTCGGACTGTAGAACTCTGAAC
FBC_Oligo77	CAGGTCACTAGCGTGATCGTCGGACTGTAGAACTCTGAAC
FBC_Oligo78	CAGGTCCGTTCGTTGATCGTCGGACTGTAGAACTCTGAAC
FBC_Oligo79	CAGGTCAGTCACGAGATCGTCGGACTGTAGAACTCTGAAC
FBC_Oligo80	CAGGTCCCTGTAACGATCGTCGGACTGTAGAACTCTGAAC
FBC_Oligo81	CAGGTCGTCCTCTTGATCGTCGGACTGTAGAACTCTGAAC
FBC_Oligo82	CAGGTCCAGCGAATGATCGTCGGACTGTAGAACTCTGAAC
FBC_Oligo83	CAGGTCATGGTTGGGATCGTCGGACTGTAGAACTCTGAAC
FBC_Oligo84	CAGGTCGAGGTTCTGATCGTCGGACTGTAGAACTCTGAAC
FBC_Oligo85	CAGGTCTACCTCGAGATCGTCGGACTGTAGAACTCTGAAC
FBC_Oligo86	CAGGTCTTCTGTGCGATCGTCGGACTGTAGAACTCTGAAC
FBC_Oligo87	CAGGTCGACAACTGGATCGTCGGACTGTAGAACTCTGAAC
FBC_Oligo88	CAGGTCCGACAACAGATCGTCGGACTGTAGAACTCTGAAC
FBC_Oligo89	CAGGTCTCGATACCGATCGTCGGACTGTAGAACTCTGAAC
FBC_Oligo90	CAGGTCCCATACTCGATCGTCGGACTGTAGAACTCTGAAC
FBC_Oligo91	CAGGTCATTCGCAGGATCGTCGGACTGTAGAACTCTGAAC
FBC_Oligo92	CAGGTCACCATAGGGATCGTCGGACTGTAGAACTCTGAAC
FBC_Oligo93	CAGGTCCGATCAAGGATCGTCGGACTGTAGAACTCTGAAC
FBC_Oligo94	CAGGTCACCTTGCTGATCGTCGGACTGTAGAACTCTGAAC
FBC_Oligo95	CAGGTCGACTCAGTGATCGTCGGACTGTAGAACTCTGAAC
FBC_Oligo96	CAGGTCGTCAATCCGATCGTCGGACTGTAGAACTCTGAAC

Table 6. Oligonucleotide sequences used to generate the second set of barcoded beads (SBC) for combinatorial synthesis in Experiment 2.

Oligonucleotide Name	Oligonucleotide Sequence
SBC_Oligo1	AAAAAAAAAAAAAAAAAAAAAAAAAATATGCGCAGGTCAAAAAAAAAGATCGTCGGACTGTAGAACTC
SBC_Oligo2	AAAAAAAAAAAAAAAAAAAAAAAAAAGGACATCAGGTCAAAAAAAAAGATCGTCGGACTGTAGAACTC
SBC_Oligo3	AAAAAAAAAAAAAAAAAAAAAAAAAGACTACGCAGGTCAAAAAAAAAGATCGTCGGACTGTAGAACTC
SBC_Oligo4	AAAAAAAAAAAAAAAAAAAAAAAAACTGAAACCAGGTCAAAAAAAAAGATCGTCGGACTGTAGAACTC
SBC_Oligo5	AAAAAAAAAAAAAAAAAAAAAAAAATAGGACCCAGGTCAAAAAAAAAGATCGTCGGACTGTAGAACTC
SBC_Oligo6	AAAAAAAAAAAAAAAAAAAAAAAAATAACGCACAGGTCAAAAAAAAAGATCGTCGGACTGTAGAACTC
SBC_Oligo7	AAAAAAAAAAAAAAAAAAAAAAAAACCCAACACAGGTCAAAAAAAAAGATCGTCGGACTGTAGAACTC
SBC_Oligo8	AAAAAAAAAAAAAAAAAAAAAAAAACGCATTTCAGGTCAAAAAAAAAGATCGTCGGACTGTAGAACTC
SBC_Oligo9	AAAAAAAAAAAAAAAAAAAAAAAAACATCTACCAGGTCAAAAAAAAAGATCGTCGGACTGTAGAACTC
SBC_Oligo10	AAAAAAAAAAAAAAAAAAAAAAAAATACGGATCAGGTCAAAAAAAAAGATCGTCGGACTGTAGAACTC

Table 7. Table of reagents and cost estimates per run. Costs associate with combinatorial bead synthesis are highlighted in gray.

Reagent	Volume	Stock Volume	Price of Stock	Price per Run
SUPERaseIN (Ambion)	19 uL	500 uL	$350.40	$13.32
dNTPs (NEB)	10 uL	800 uL	$44.80	$0.56
HiScribe IVT Kit (NEB)	1 uL	50 uL	$169.60	$3.39
MessageAamp II Kit (Ambion)*	3 uL	740 uL	$3,668.00	$14.87
PrimeScript RT (Clontech)	5 uL	200 uL	$501.63	$12.54
Phusion polymerase (NEB)	0.5 uL	250 uL	$336.00	$0.67
Lane Barcode RT primers (IDT)	15 uL	3155 uL	$542.25	$0.86
NHS beads (GE)	3.25 uL	25000 uL	$155.80	$0.02
Streptavidin (NEB)	5 uL	1000 uL	$188.80	$0.94
Dual-biotin anchor oligo (IDT)	0.64 uL	700 uL	$225.75	$0.21
Klenow fragment exo- (NEB)	1.5 uL	200 uL	$188.80	$1.42
dNTPs (NEB)	3.5 uL	800 uL	$44.80	$0.20
FBC primers (IDT)	0.96 uL (all 96)	240,000 uL (all 96)	$484.56 total	$0.002
SBC primers (IDT)	0.66 uL (all 10)	4000 uL (all 10)	$340.00 total	$0.67
**Experiment Costs**				**$46.21**
**Bead Costs**				**$3.46**
**Total Cost per Run**				**$49.67**
**Cost per Cell**	**250-500 cells**			**$0.10-$0.20**

* Only the second-strand synthesis reagents are used here.
